# Multidimensional Measurement of Household Water Poverty in a Mumbai Slum: Looking Beyond Water Quality

**DOI:** 10.1371/journal.pone.0133241

**Published:** 2015-07-21

**Authors:** Ramnath Subbaraman, Laura Nolan, Kiran Sawant, Shrutika Shitole, Tejal Shitole, Mahesh Nanarkar, Anita Patil-Deshmukh, David E. Bloom

**Affiliations:** 1 Partners for Urban Knowledge, Action, and Research (PUKAR), Mumbai, India; 2 Division of Infectious Diseases, Brigham and Women’s Hospital and Harvard Medical School, Boston, MA, United States of America; 3 Office of Population Research, Woodrow Wilson School of Public and International Affairs, Princeton University, Princeton, NJ, United States of America; 4 Department of Global Health and Population, Harvard School of Public Health, Boston, MA, United States of America; The Foundation for Medical Research, INDIA

## Abstract

**Objective:**

A focus on bacterial contamination has limited many studies of water service delivery in slums, with diarrheal illness being the presumed outcome of interest. We conducted a mixed methods study in a slum of 12,000 people in Mumbai, India to measure deficiencies in a broader array of water service delivery indicators and their adverse life impacts on the slum’s residents.

**Methods:**

Six focus group discussions and 40 individual qualitative interviews were conducted using purposeful sampling. Quantitative data on water indicators—quantity, access, price, reliability, and equity—were collected via a structured survey of 521 households selected using population-based random sampling.

**Results:**

In addition to negatively affecting health, the qualitative findings reveal that water service delivery failures have a constellation of other adverse life impacts—on household economy, employment, education, quality of life, social cohesion, and people’s sense of political inclusion. In a multivariate logistic regression analysis, price of water is the factor most strongly associated with use of inadequate water quantity (≤20 liters per capita per day). Water service delivery failures and their adverse impacts vary based on whether households fetch water or have informal water vendors deliver it to their homes.

**Conclusions:**

Deficiencies in water service delivery are associated with many non-health-related adverse impacts on slum households. Failure to evaluate non-health outcomes may underestimate the deprivation resulting from inadequate water service delivery. Based on these findings, we outline a multidimensional definition of household “water poverty” that encourages policymakers and researchers to look beyond evaluation of water quality and health. Use of multidimensional water metrics by governments, slum communities, and researchers may help to ensure that water supplies are designed to advance a broad array of health, economic, and social outcomes for the urban poor.

## Introduction

Approximately 863 million people globally live in urban slums [1]. Inadequate access to water is one characteristic that helps to define a “slum,” based on the United Nations (UN) definition [2, 3]. Most public health studies of water service delivery in slums have had a somewhat limited scope of investigation, as they focus on water quality as the primary indicator of interest, due to associations with health outcomes, especially diarrheal illness [4–11].

Questions remain, however, regarding the relative importance of quality as compared with other water service indicators for slum populations. In rural settings, the prevalence of unimproved water supplies makes bacterial contamination an issue of central importance. In contrast, studies of water quality in slums suggest that point-of-source bacterial contamination may be less common, especially when water is obtained from taps, because many city water supplies are centrally chlorinated [6, 7, 10, 11]. While slum residents are often exposed to point-of-use contamination from unsafe water storage [4–6, 8–10], the contribution of such household-level contamination to health outcomes remains unclear [8]. Few studies in slums have evaluated other water service indicators (e.g., quantity, reliability, or access) or non-health-related outcomes resulting from inadequate service delivery (e.g., economic or quality-of-life outcomes) [12–16].

In this mixed methods study of Kaula Bandar (KB), a slum in Mumbai, we look beyond water quality to illuminate the importance of other water service delivery indicators and to characterize adverse economic, social, and health impacts resulting from inadequate water supply. Notably, we define “water service delivery” to encompass not only formal water supply by governments, but also the diverse informal processes of procurement, household storage, and water consumption that occur in slums. We build upon a prior study of KB’s informal water distribution system, which focused on evaluating water quality [10]. We emphasize understanding use of an inadequate water *quantity*, as this indicator may account for considerable variability in health outcomes [17].

We first use the qualitative data to illuminate the adverse life impacts KB’s residents face due to deficiencies in water service delivery. We then perform a multivariate logistic regression analysis of quantitative data collected during a survey of 521 households to identify predictors associated with use of an inadequate quantity of water. We further analyze these quantitative data to understand the trade-offs KB residents face in choosing to use the different modes of water access available in the slum.

We integrate these findings to propose a multidimensional framework for defining and assessing household-level “water poverty.” This framework encourages researchers to look beyond evaluation of water quality alone, in favor of investigating a broader constellation of water service indicators and associated health, economic, and social outcomes in future studies of water supply in slums. Finally, we discuss the potential benefits for governments and slum communities of using this multidimensional approach to assessing water poverty.

## Methods

### Study site

KB is a community of about 12,000 people located on a dock on Mumbai’s eastern waterfront. KB was first settled decades ago by immigrants from Tamil Nadu who worked on Mumbai’s port; as the port has slowly gone defunct, many Tamilians now work in a nearby shipbreaking industry or as garbage collectors. In the last two decades, the slum has seen the arrival of immigrants from Uttar Pradesh, many of whom run small-scale industries, making belts, bags, or clothing.

As KB is “non-notified,” or not recognized by the city, state, or central governments, the community is denied legal access to most public services, including electricity, sanitation infrastructure, and the city water supply. Because sewage infrastructure was never created, in-home toilets are virtually nonexistent. KB has a few pay-for-use toilet blocks with only eight functional seats at the time of our study. A substantial proportion of adults engage in open defecation (S1 Appendix, Table A). KB has higher infant mortality, child undernutrition, and illiteracy rates than most other slums in Mumbai [18].

### Water access in Kaula Bandar

There are two main modes of water access in KB. Both rely on surreptitious procurement of water from the city supply, because residents do not have legal access. About two-thirds of households purchase water through an informal distribution system run by private vendors, most of whom are KB residents (S1 Appendix, Table A). These vendors tap into underground city water pipes and use motorized pumps to funnel water into hoses that travel hundreds of meters to reach slum lanes. Residents pay monthly fees to access water from these hoses. Due to the intermittent nature of water flow, water is available for two hours a day at most, so residents line up in lanes at water delivery times to access a hose. Hereafter, these households will be referred to as *hose water recipients*.

One-third of households do not obtain water through the informal distribution system. Instead, they roll containers long distances (often more than a kilometer) to purchase water from taps located in other slums. They pay the tap’s “owners” (usually occupants of a nearby household) per container filled. Hereafter, these households will be referred to as *water fetchers*. The reasons why households choose to be hose water recipients or water fetchers are discussed below.

The informal distribution system is precarious; every few months, government officials raid and confiscate the motorized pumps en masse. During these periods of “system failure,” the distinction between hose water recipients and water fetchers breaks down, and everyone gets water by rolling containers to taps outside of KB. Less commonly, expensive private tankers are hired to bring water. We collected data for the current study when the informal distribution system was functional. As such, the findings reflect the “normal” division between hose water recipients and water fetchers. The quantitative data were collected during Mumbai’s “winter” season and therefore do not reflect seasonal variation in water indicators, which we highlighted in a prior study [10].

### Ethical review and informed consent

The PUKAR Institutional Ethics Committee (FWA00016911) approved the protocol for both the qualitative and quantitative study phases in February 2011. A verbal informed consent process was used for the qualitative focus group discussions (FGDs) and individual interviews and for the structured survey questionnaire in the quantitative phase of the study. The PUKAR Institutional Ethics Committee approved all verbal informed consent protocols and forms.

We chose to use verbal informed consent due to the low literacy level in KB, which means that some residents have difficulty signing their names. While we could have obtained each study participant’s thumbprint instead of his or her signature, we found in previous research that many KB residents are hesitant to provide a thumbprint even when they are agreeable to participating in a study, as they associate thumbprints with government bureaucratic procedures.

The researcher read a scripted consent form describing the purpose of the study, emphasizing that the participant could choose to discontinue the interview at any time. The participant was given time to ask questions about the study. If he or she agreed to participate, the researcher signed the form and recorded the participant’s name to indicate consent. A copy of the consent form, which included contact information for the principal investigators at PUKAR, was provided to all study participants, and they were encouraged to contact PUKAR with any concerns. All research personnel, including the “barefoot researchers,” underwent ethics training prior to conducting the study.

KB is located on land owned by the government. Because the land is public, no permissions were required to conduct this research. The field research did not involve endangered or protected species.

### Qualitative data collection

The qualitative and quantitative data were collected as part of a larger investigation of mental health in KB; gathering data on water poverty was a secondary study objective. We describe the methods in brief, focusing on relevant water-related aspects, as a detailed description of the study approach is available in a prior manuscript [19].

Collection, analysis, and reporting of the qualitative data follow the Consolidated Criteria for Reporting Qualitative Research (COREQ) 32-item checklist [20]. For the qualitative research phase, individuals older than 18 years of age were purposefully sampled using a framework that reflects KB’s gender, religious, and ethnic diversity. In July 2011, four of the authors (TS, KS, SS, RS) conducted three FGDs with women (age range 22–65, median 35 years) and three FGDs with men (age range 19–70, median 30 years), all of which had six to nine participants. Participants were approached and recruited from their homes and the FGDs took place in a private office space in the slum.

In November 2011, four of the authors (TS, KS, SS, RS) collected 20 individual interviews with women (age range 21–55, median 33.5 years), 19 with men (age range 25–72, median 38 years), and one with a transgender resident (age 48 years). Participants were approached and recruited from their homes, and most interviews took place in the participants’ homes. All women’s interviews and most of the men’s interviews were conducted by a researcher of the same gender.

Notably, two of the interviewers are female and two are male. All interviewers were research associates at PUKAR at the time of the study. Three of the interviewers (TS, KS, SS) have at least a Bachelor’s or a Master’s degree and had spent four years conducting field research in KB prior to this study. One (RS) is a medical doctor with one and a half years of prior research experience in KB. Given the longstanding relationship between the researchers and the community, most study participants were aware of PUKAR’s larger goal of conducting an interdisciplinary study of health in KB.

The qualitative questionnaire consisted of an open-ended exercise in which respondents were asked to describe adversities they face from living in KB. Researchers were given an interview guide that had undergone extensive pretesting to facilitate the FGDs and interviews. Water-related hardship was a dominant topic that arose in every interview and FGD. All interviews or FGDs were audio-recorded over 40–60 minutes and were conducted in Hindi, Tamil, or Marathi, after obtaining verbal informed consent as described previously.

### Qualitative data analysis

We reviewed all FGD and interview transcripts using an inductive approach to analysis based in grounded theory [21]. One of the authors (RS) read all interview transcripts and extracted all relevant water-related quotations. We then coded each individual quotation for the following: 1) the *water service delivery failure/s* being described in the quotation and 2) the *adverse life impacts* that result from deficiencies in water service delivery. Our goal was to identify the adverse life impacts resulting from the water situation in KB and their association with specific water service delivery failures.

To understand water service delivery failures, we coded each quotation using the following indicators, which were identified based on existing and emerging water frameworks [22, 23]: quality, quantity, access, reliability, price (or affordability), and equity. Subjective negative perceptions of the appearance, smell, taste, or perceived microbiological status of water were classified as problems of *quality*. Difficulties arising from having an inadequate amount of water were classified problems of *quantity*. Difficulties arising from the distance from water sources or time spent collecting water were classified as problems of *access*. Difficulties arising from intermittent water delivery or uncertainty around water timings were classified as problems of *reliability*. Difficulties in paying for water, or negative impacts of water spending on purchasing food or other necessities, were classified as problems of *price or affordability*. Finally, perceptions of discrimination in water access or price relative to other households in KB or other city dwellers were classified as problems of *equity*. These indicators are often interrelated—for example, problems of access or reliability may result in inadequate water quantity. Therefore, many quotations were classified as expressing deficiencies in multiple water service delivery indicators.

To understand the adverse life impacts resulting from KB’s water situation, we first conducted a careful reading of the water-related quotations. Using an inductive approach, we identified the following aspects of life that are adversely impacted by deficiencies in water service delivery: household economy, employment, education, quality of life, social relationships, community cohesion, social and political inclusion, and health. Each quotation was then coded using these categories.

Finally, to understand the relationship between water service delivery failures and adverse life impacts, we created a “concept map” that diagrams these relationships based on the coding for each quotation [24].

### Quantitative data collection

**Fig 1:**
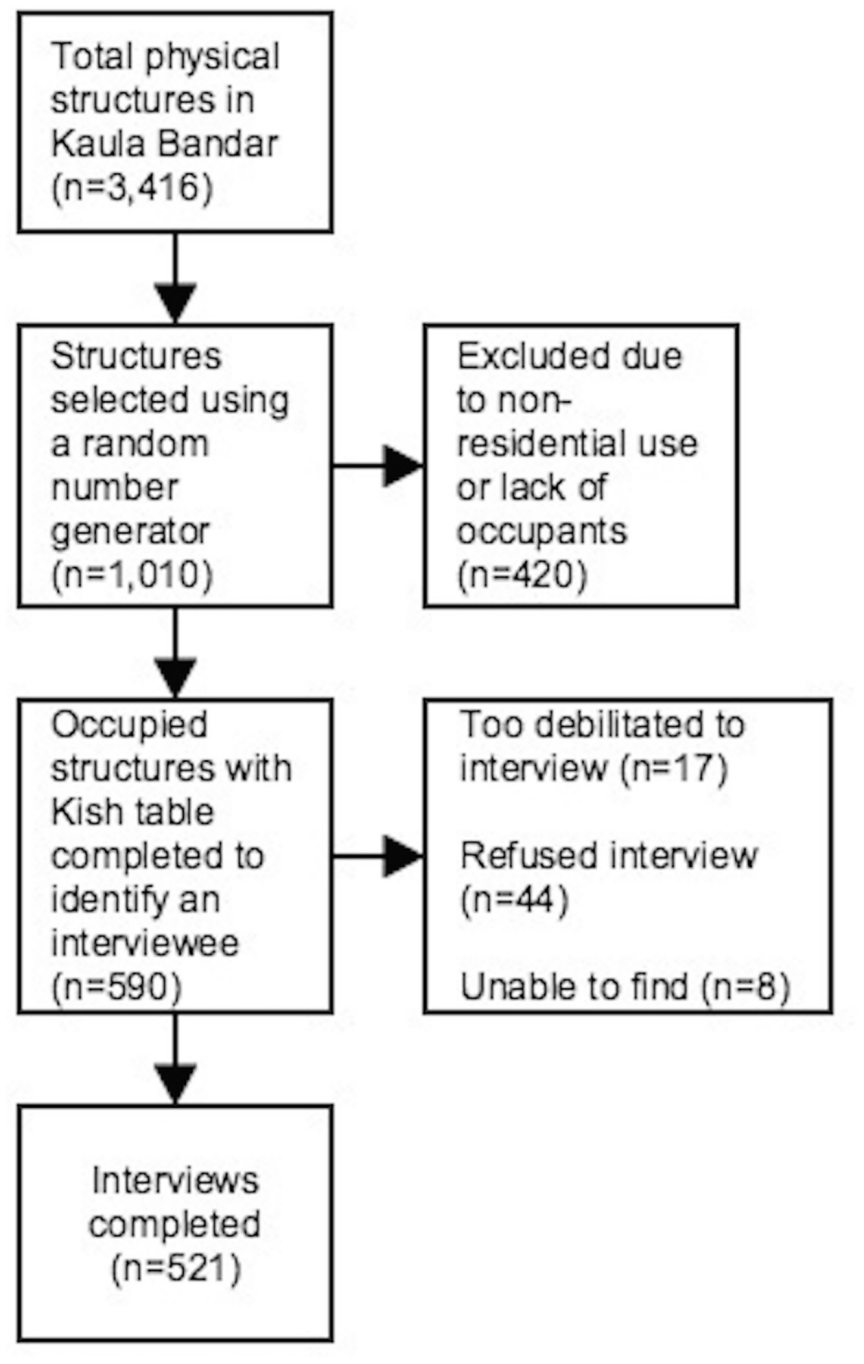
The sample selection process for the quantitative survey.

For the quantitative phase, we used a structured questionnaire, which took 30–40 minutes to administer, with respondents selected using population-based random sampling of households (Fig 1). The questionnaire was administered after obtaining informed consent as described previously. We enumerated 3,416 potential living structures in KB in January 2012. Using a random number generator, we sampled 1,010 structures from this database. We eliminated 420 of these structures, as they were being used for nonresidential purposes or were found to be unoccupied after a minimum of three visits. For the remaining households, we used a Kish table to objectively select a respondent over 18 years of age [19]. PUKAR’s barefoot researchers (BRs)—local youths with at least a high school education, most of whom live in KB—administered surveys in February 2012. Excluding 17 individuals who were unable to answer the questionnaire due to severe illness or disability, we achieved a 9% non-response rate.

Because hose water recipients pay the water vendors monthly, assessing their monthly water spending was easy. In contrast, water fetchers pay per container of water filled; however, most women have an excellent sense of weekly household water spending. From this estimate of weekly spending, BRs estimated monthly spending by multiplying by four. Notably, despite the necessity of using different approaches to estimate water spending, the median price of water is similar for the two groups, providing confidence in the credibility of these data (see the section below entitled “Trade-offs in modes of water access”).

To assess water quantity used, we trained BRs to estimate the volume of water held by containers of different sizes commonly used to store water in KB. After listing each container in the household, BRs asked respondents about the number of times each container had been filled with water in the last week. The estimated volume of each container was multiplied by the number of times it had been filled in the last week to estimate total weekly household water use. For interested readers, the S2 Appendix compares PUKAR’s method for measuring water quantity with methods used in other studies, and the S3 Appendix presents the full study questionnaire.

### Quantitative analysis

We analyzed the quantitative data using STATA version 13 (College Station, TX, U.S.A.) and created graphs using R version 3.03 (Vienna, Austria). To identify factors associated with the dependent variable of inadequate water use (≤20 liters per capita per day or LPCD), we fit a multivariate logistic regression model with predictors including demographic and water-related variables. To identify factors associated with water quantity consumed as a continuous outcome, we also fit a multivariate ordinary least squares (OLS) regression model. To calculate household income per capita, we weighted each child <14 years of age as 0.5 of an adult, because child costs to range from 48–60% of adult costs in Indian studies [25].

We assessed the relationship between the average price paid for water by a household per 1,000 liters of water (hereafter “price of water”) and quantity of water using a scatterplot. We regressed quantity of water in natural logarithms on the price of water in natural logarithms and took the associated β-coefficient as an estimate of price elasticity. To understand inequities in water-related hardship within KB, we calculated Gini coefficients and created histograms depicting the distribution of household income per capita, quantity of water used, price of water, and monthly water spending as a percentage of household income. We tested for statistically significant differences between characteristics of the hose water recipients and water fetchers using the Chi-squared test for categorical variables and the Wilcoxon test for continuous variables. The quantitative dataset contains potentially identifying information regarding study participants. Due to ethical restrictions, the quantitative dataset is available to interested readers upon request from the corresponding author, pending ethical approval.

## Qualitative Results

Table 1 presents categories of adverse life impacts that KB residents experience and the water service delivery indicators contributing to these impacts, based on the qualitative data (S1 Appendix, Table B, presents a more detailed analysis). Water service delivery failures have a constellation of negative impacts—on household economy, employment, education, health, quality of life, social relationships, community cohesion, and people’s sense of political inclusion.

**Table 1:** Adverse life impacts of deficiencies in water service delivery in Kaula Bandar based on analysis of the qualitative data.

Adverse life impact	Water service delivery failures causing the adverse life impact	Representative quotations
1. Household economy	price, quantity	“We need money for everything. For water, we have to buy it every day… How can I manage everything? Every day we need to decide whether we want food or water.”
		*-30-year-old Muslim woman from Uttar Pradesh*
		“We take out loans sometimes; just when we finish repaying those loans, we often need to take out another loan. I spend a lot of money on purchasing water.”
		*-55-year-old Hindu woman from Tamil Nadu*
2. Employment	access, reliability, quantity	“My boss called me today, but I didn’t go to work because I had to fill water this evening. There is no water in my home.”
		*-20 year old Muslim man from Uttar Pradesh*
		“I work at a call center. Our call center rule is that you always have to look fresh. Sometimes I skip work because there is no water to take a bath.”
		*-25-year-old Muslim man from Uttar Pradesh*
3. Education	quantity, access, reliability	“How can she get an education if there is no water in our area! People are always sending their children to bring water. Because they fill water for their homes, they never go to school.”
		*-40-year-old Hindu man from Tamil Nadu*
		“Teachers blame us for sending our children to school without giving them a bath. They don’t understand the problems we face here.”
		*-33-year-old woman from Tamil Nadu in a women’s focus group discussion*
		“My child failed his last three assignments in math, English, and Marathi, because he was always going back and forth to get water. Because of our water crisis, our children are losing their education. One of my sons had an economics paper to do, but he spent all day filling water, and that’s why he failed on that paper.”
		*-40-year-old Muslim woman from Uttar Pradesh*
4. Health	quantity, quality, reliability, access	“Sometimes we don’t get water for a week. I can’t clean my home because I don’t have enough water, so my home stinks. I don’t like it, but I have no choice. There is not enough water to bathe my children. If guests come to my home then I am embarrassed. I know how important it is to clean, but I can’t clean my clothes and utensils properly. And then we get sick. I know all these things, but I don’t know how to deal with this problem.”
		*-35-year-old Hindu woman from Maharashtra*
		“People who live here, they often don’t get drinking water for more than a week. Sometimes bugs grow in our drinking water containers, and that’s why people get sick.”
		*-26-year-old Hindu man from Maharashtra*
5. Quality-of-life	quantity, reliability, access	“Suppose water doesn’t come for 10 days. After five days, we begin to run out of water, and I can’t complete any of my housework. During those times, my main preoccupation is wondering when water will come again. All of our clothes accumulate in the house, because we can’t wash them. If we had water, we could actually complete our daily household chores. During times when water doesn’t come, I have a lot of tension. At some point, even cooking food becomes difficult. In the end, we always have to set aside some water to make sure that we have enough to drink, even if we aren’t washing the clothes or the dishes or cooking… Water usually comes to us again after one week. If water doesn’t come after more than a week, it’s unbearable.”
		*-30-year-old Hindu woman from Tamil Nadu*
		“We have to push the water drum all the way from our home to beyond the police station [outside of Kaula Bandar] where the taps are. Now that we’re getting old, it is difficult… So most of the time we don’t take the big drums to get water, and we just carry water in small containers on our heads.”
		*-48-year-old Hindu woman from Tamil Nadu*
		“Last summer, we were staying up all night just filling water. No one delivers water to my home.”
		*-42-year-old Muslim man from Uttar Pradesh*
		“Sometimes the water comes at 2 AM, 3 AM, or 4 AM, but whenever it comes we have to be there waiting for it. It doesn’t run on any timetable.”
		*-30-year-old Hindu man from Tamil Nadu*
6. Social relationships	quantity, reliability, access	**“**Suppose our relatives come from our native villages in Tamil Nadu. In their homes, they have their own bathrooms and they have many amenities. But during the 10 days they spend here, they are shocked at how hard it is to go to the bathroom and get water. So then they leave early. And they always say to me, ‘Things are so easy where we live, it’s easy for us to get water.’ They usually leave within four days.”
		*-48-year-old Hindu woman from Tamil Nadu*
7. Community cohesion / political inclusion	equity, quantity, reliability, access, price	“Ramzan [Ramadan] is a big festival for us. But during that festival, sometimes we don’t get water. So we can’t keep ourselves clean.”
		*-40-year-old Muslim woman from Uttar Pradesh*
		“The water mafia [informal water vendors] run their own system here. The government doesn’t help us. The politicians tell us, ‘We will help you,’ but then they do nothing… The water mafia charges whatever price they want. They only give water once a week. We can’t speak out against them, because they are thugs.”
		*-38-year-old Christian man from Kerala*
		“In the past, a politician came and placed a water pipe around the time of an election. After the election, he just left the work as it was without completing it. That pipe is still sitting in the ground, useless. He won the election and then just forgot about us. Even now they’re digging up the road, placing a new pipe for the next election.”
		*-30-year-old Hindu man from Tamil Nadu*
		“We have this water crisis primarily because we are on [Mumbai Port Trust] land. I feel like they are stopping the water because they eventually want us to leave this land.”
		*-32-year-old Christian woman from Tamil Nadu*

Concerning household economy, water spending consumes a substantial proportion of the household budget, cutting into food spending and sometimes resulting in residents taking out loans. Problems with access and reliability result in missed days of work or in residents leaving jobs to fetch water; women’s domestic labor, such as childcare duties, is also adversely affected. Children are late for, or miss, school due to water collection duties; these duties also compromise time available for homework. Children and adults sometimes miss school or work as a result of embarrassment over body odor, due to lack of water for bathing.

With regard to health, some residents express concerns that poor water quality (e.g., foul-smelling or visibly dirty water) may be associated with illness. Most of these residents express concerns about post-source contamination, as water fetchers often have to roll their water drums through a large trash dump on KB’s main road, and hose water recipients store water for days in unsafe (wide-mouthed) containers. Many residents also believe that adverse health impacts result from inadequate water quantity, which leads to deterioration of hygiene in public spaces; the home; and, most crucially, public toilets, which also lack water supplies.

With regard to quality-of-life, water fetchers, especially the elderly, express concerns about the physical challenges of water collection. Some describe spending the whole night collecting water. Hose water recipients lose sleep due to the unpredictable nature of water flows, which sometimes arrive in the middle of the night. Women express distress over the inability to complete chores—washing clothes, cleaning dishes, cooking—because of insufficient water quantity.

With regard to social relationships, residents describe how relatives visiting from rural villages return early due to lack of water. Water fetchers get into conflicts with neighbors when rolling water drums through shared lanes. Community cohesion is compromised when festivals turn into crises because of lack of water. Residents harbor resentment against water vendors, who engage in price gouging, and against government officials, who periodically raid vendors’ motors and shut down the water supply. All of these service delivery failures—and lack of provision of any formal water supply by the government over decades—lead to cynicism about the democratic process, compromising residents’ sense of political inclusion in the life of the larger metropolis.

**Fig 2:**
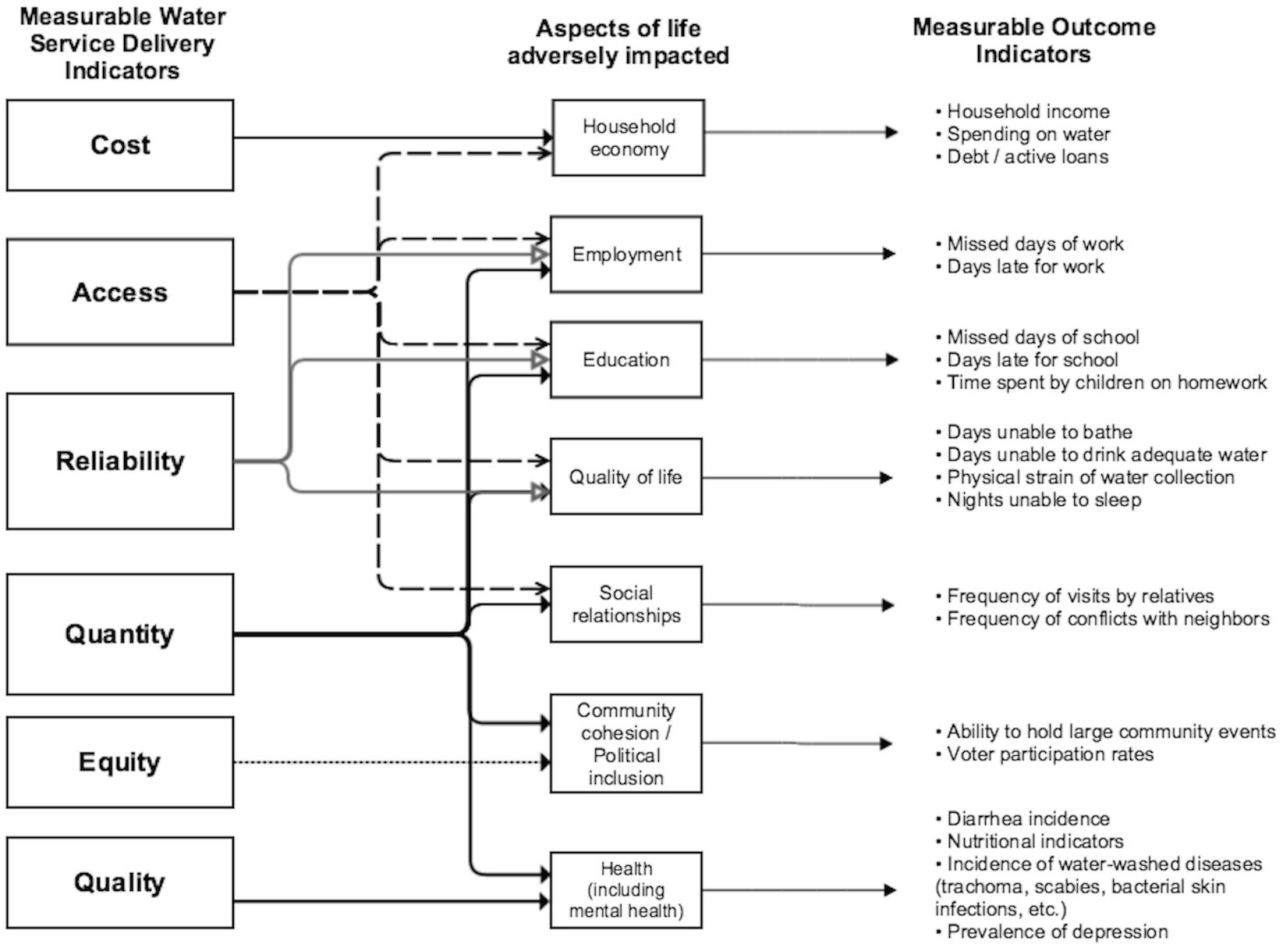
The relationships among water service delivery failures and adverse life impacts based on the qualitative findings. This diagram may also serve as a multidimensional framework for defining and evaluating household-level “water poverty” in slums.

Based on the qualitative analyses presented in Table 1, we created a concept map (Fig 2) that diagrams the relationships among water service delivery failures and adverse life impacts.

KB residents described deficiencies in multiple water service delivery indicators—quantity, quality, affordability, reliability, access, and equity—which are represented on the left side of the concept map in Fig 2. In turn, as shown in the central part of Fig 2, these water service delivery failures map onto multiple aspects of life that are adversely affected—including health, the household economy, employment, education, social relationships, community cohesion, and political inclusion. In the right side of Fig 2, we propose possible “outcome indicators” for measuring these adverse life impacts resulting from an inadequate water supply.

## Quantitative Results

### Descriptive characteristics

S1 Appendix, Table A, presents the demographic composition and water indicators for the 521 households included in the quantitative survey. The median water quantity use is 23 LPCD. On average, households spend 9.5% of their monthly household income on water. More than one-fourth of households obtained water only once in the prior week, highlighting poor reliability.

**Fig 3:**
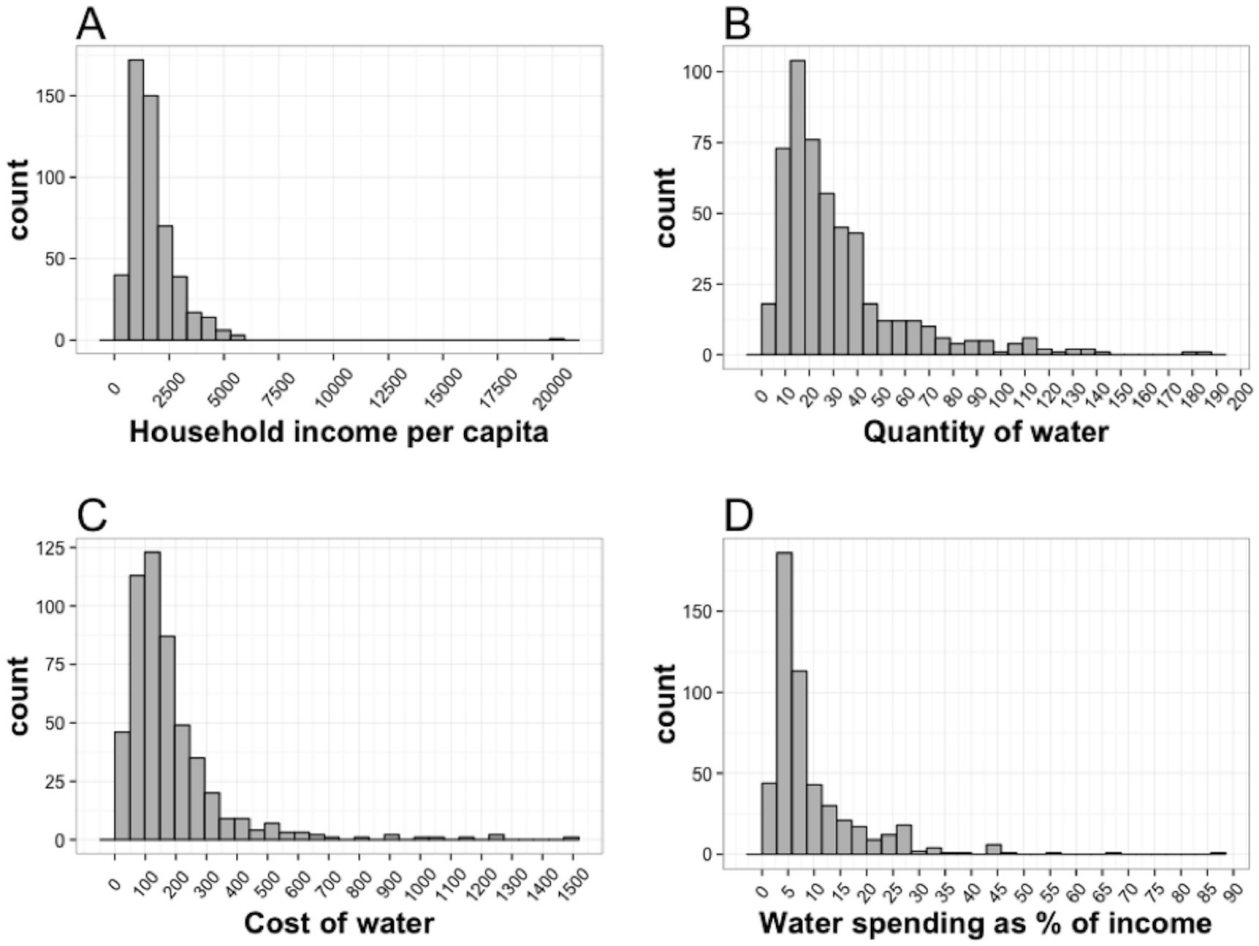
The distribution of water-related indicators in Kaula Bandar. (A) Household income per capita in the last month in Indian rupees (Gini coefficient = 0.31); (B) quantity of water consumed in liters per capita per day (Gini coefficient = 0.42); (C) price of water in Indian rupees per 1,000 liters of water (Gini coefficient = 0.41); and (D) water spending as a percentage of household income in the last month (Gini coefficient = 0.47).

To better understand water equity in KB, we calculated Gini coefficients for multiple water indicators, with 0 representing perfect equality and 1 representing perfect inequality (Fig 3). Inequality in household income is moderate, with a Gini coefficient of 0.31. Inequality is greater in water service indicators, with Gini coefficients of 0.41, 0.42, and 0.47 for water price, quantity of water used, and water spending as a percentage of household income, respectively.

For comparison, in flats (apartments) in Mumbai with formal meters, the city government currently charges a standard price of Indian rupees (INR) 5 per 1,000 liters of water (as of 2013). Therefore, we expect a Gini coefficient of 0 for Mumbai’s formally housed population, because water charges are consistent across all households. For residents in notified (“legal”) slums in Mumbai the city government charges a standard price of INR 3 per 1,000 liters of water; however, because water in notified slums is distributed through community taps, we anticipate that price inequality may still exist among these households, though probably less than the price inequality in KB. The degree of inequality in water quantity and water spending for formal flats and notified slum dwellers is unclear as data on these indicators are not are available for these populations from prior studies.

### Predictors of using an inadequate quantity of water

In the multivariate logistic regression model, paying a high price for water, having more than three people in the household, and renting one’s home are associated with an increased risk of using ≤20 LPCD (Table 2). Engaging in water fetching, getting water more than once a week, and having higher income per capita protect against use of ≤20 LPCD. Price of water has the most substantial association with use of an inadequate water quantity.

**Table 2:** Predictors of inadequate water quantity in a multivariate logistic regression model.

Risk factor	Severe inadequacy of water use (≤20 liters per capita per day)	Univariate findings	Multivariate findings (N = 508, pseudo-*R* ^2^ = 0.467)	
	*N (%)*	*Odds ratio (p-value)*	*Odds ratio (CI)*	*p-value*
**Number of people in household**				
< = 3	34(25.8)	-	-	-
4–5	86(42.0)	2.08(0.003)	4.35(1.98–9.57)	<0.001*
> = 6	103(56.0)	3.67(<0.001)	15.70(5.88–41.95)	<0.001*
**Religion**				
Hindu	120(47.1)	-	-	-
Muslim	93(38.6)	0.71(0.057)	0.92(0.49–1.73)	0.803
Christian or Buddhist	10(40.0)	0.75(0.50)	1.11(0.28–4.36)	0.883
**Region of origin**				
North Indian	120(48.8)	-	-	-
South Indian	54(32.9)	0.51(0.002)	0.60(0.28–1.31)	0.199
Maharashtrian	34(42.5)	0.78(0.329)	0.62(0.27–1.40)	0.254
Nepali, other	15(48.4)	0.98(0.967)	1.60(0.49–5.21)	0.433
**Home ownership status**				
Owns his/her living space	120(36.8)	-	-	-
Rents his/her living space	103(52.8)	1.92(<0.001)	2.10(1.10–3.83)	0.023*
**Household monthly income per capita**				
INR^a^<1,000	38(60.0)	-	-	-
INR 1,000–1,499	65(42.8)	0.51(0.001)	0.44(0.20–0.98)	0.044*
INR 1,500–1,999	46(40.0)	0.44(0.004)	0.80(0.34–1.89)	0.615
INR 2,000–2,499	23(45.1)	0.56(0.096)	0.70(0.24–2.09)	0.528
INR 2,500+	29(29.3)	0.28(0.086)	0.29(0.10–0.82)	0.020*
**Price of water**				
INR <100 per 1,000 liters	19(11.6)	-	-	
INR 100–199 per 1,000 liters	93(44.5)	6.12(<0.001)	7.93(3.75–16.77)	<0.001*
INR 200+ per 1,000 liters	111(75.0)	22.89(<0.001)	57.42(23.22–141.96)	<0.001*
**Method of obtaining water**				
Water delivered	49(27.4)	-	-	-
Carries containers a distance to fetch water	173(51.0)	0.36(<0.001)	0.24(0.12–0.45)	<0.001*
**Number of times in a week water was obtained**				
1 time	120(81.1)	-	-	-
2–3 times	94(32.5)	0.11(<0.001)	0.17(0.09–0.33)	<0.001*
4 or more times	9(10.8)	0.03(<0.001)	0.04(0.01–0.12)	<0.001*
**Model constant**	-	-	0.19(0.04–0.88)	<0.034*

^a^INR = Indian rupees

In the multivariate OLS regression model, in which water quantity is a continuous outcome, the findings are qualitatively similar, with the exception that South Indian ethnicity is also significantly associated with higher water use (S1 Appendix, Table C).

**Fig 4:**
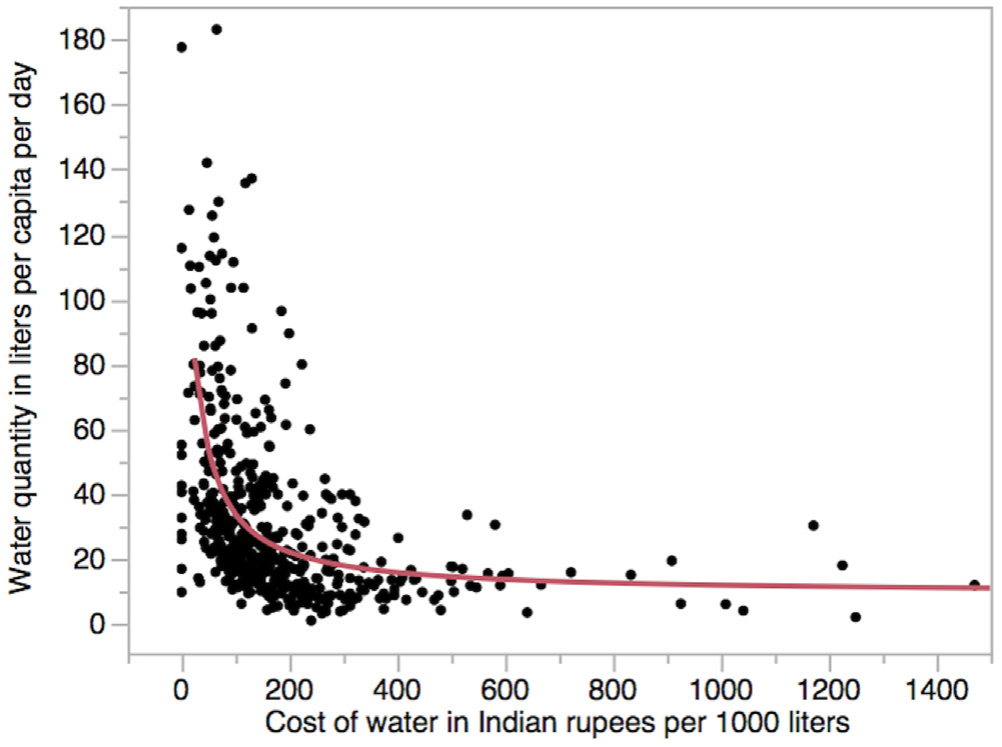
The relationship between price of water and quantity of water consumed.

We created a scatterplot to further explore the relationship between water quantity used and price of water, given the strong association between these variables in the multivariate models (Fig 4). Below a price of about INR 400 per 1,000 liters of water, water quantity used by households is sensitive to price, increasing in a non-linear fashion as price decreases. Above INR 400 per 1,000 liters, water use does not drop much below 15 LPCD, suggesting that people will pay nearly anything to maintain this basic level of water consumption. The value for elasticity is -0.6, suggesting a somewhat inelastic relationship between water quantity and price. We also produced separate scatterplots and elasticity values for the subgroups of water fetchers and hose water recipients; both were similar to the plot for the overall sample (plots not shown).

### Trade-offs in modes of water access: an illustrative example

Comparing quantitative findings between hose water recipients and water fetchers illustrates the possible benefits of a multidimensional approach to evaluating water service delivery (Table 3). Consistent with findings of the regression models, water fetchers achieve substantially higher median water use per capita and obtain water more frequently than hose water recipients.

**Table 3:** Differences in water indicators based on mode of water access.

	Water fetchers (N = 179)	Hose water recipients (N = 339)	p-value	Statistical test used
	*N (%)*	*N (%)*		
**Religion**				
Muslim	69(38.5)	184(54.3)	<0.001*	Chi-squared
Hindu	95(53.1)	145(42.8)		
Christian or Buddhist	15(8.4)	10(3.0)		
**Region of origin**				
North Indian	72(40.2)	172(50.7)	0.006*	Chi-squared
South Indian	74(41.3)	89(26.2)		
Maharashtrian	24(13.4)	56(16.5)		
Nepali	9(5.0)	22(6.5)		
**Home ownership status**				
Owns his/her living space	129(72.1)	194(57.2)	0.001*	Chi-squared
Rents his/her living space	50(27.9)	145(42.8)		
**Household monthly income per capita**				
INR^a^<1,000	43(24.2)	51(15.4)	0.002*	Chi-squared
INR 1,000–1,499	62(34.8)	87(26.3)		
INR 1,500–1,999	38(21.4)	78(23.6)		
INR 2,000–2,499	10(5.6)	41(12.4)		
INR 2,500+	25(14.0)	74(22.4)		
**Average water quantity used** in LPCD, *Median (IQR)*	31.3(18.8,55.0)	19.7(12.5,32.8)	<0.001*	Wilcoxon / Mann-Whitney
**Quantity of water used**				
Use of <20 LPCD of water	48(26.8)	171(50.4)	<0.001*	Chi-squared
Use of 20–49 LPCD	82(45.8)	137(40.4)		
Use of 50+ LPCD of water	49(27.4)	31(9.1)		
**Average water price** in INR per 1,000 liters, *Median (IQR)*	134.7(79.1,238.1)	136.6(87.7,208.3)	0.8863	Wilcoxon / Mann-Whitney
**Price of water**				
INR <100 per 1,000 liters	58(32.4)	103(30.4)	0.614	Chi-squared
INR 100–199 per 1,000 liters	67(37.4)	142(41.9)		
INR 200+ per 1,000 liters	54(30.2)	94(27.7)		
**Monthly water spending as a percentage of monthly household income**				
0–4.9%	19(10.6)	158(46.6)	<0.001*	Chi-squared
5–9.9%	70(39.1)	122(36.0)		
10–19.9%	50(27.9)	32(9.4)		
20% or above	40(22.3)	27(8.0)		
**Number of times water was obtained in the last week**				
1 time	23(12.8)	124(36.7)	<0.001*	Chi-squared
2–3 times	102(57.0)	187(55.3)		
4 or more times	54(30.2)	27(8.0)		
**Self-reported negative impact of getting water on work** (e.g., late for work, missed work, or early return from work), *N (%)*				
Yes	105(58.7)	91(26.8)	<0.001*	Chi-squared
No	74(41.3)	248(73.2)		
**Self-reported negative impact of getting water on school** (e.g., late for school, missed school, or lost time studying), *N (%)*				
Yes	43(28.5)	58(20.6)	0.0668	Chi-squared
No	108(71.5)	223(79.4)		

^a^INR = Indian rupees

At the same time, the quantitative findings reveal substantial time expenditure and opportunity costs of being a water fetcher, with 59% reporting a negative impact of water collection efforts on employment as compared with 27% of hose water recipients. On average, water fetchers spend a higher proportion of their monthly income on water. This may reflect the higher water quantity used by water fetchers (which increases overall water spending) and the lower average income for water fetchers, because the price paid for water by the two groups is similar.

In summary, either mode of water access involves considerable trade-offs. Obtaining water is a strenuous process for water fetchers, involving major commitments of income and time in a manner that negatively affects employment; however, fetching ensures more predictable water access and substantially higher water quantity per capita. In contrast, hose water recipients experience a highly unpredictable water supply, resulting in access to lower water quantities; in return, they benefit from relative ease of water access (as the hoses deliver it to their lanes), spend a smaller proportion of their income on water, and save time, which affects employment less negatively.

## Discussion

This study highlights the relevance of multidimensional assessment of water service delivery in slums. Rather than viewing water solely through the lens of quality and health, we use qualitative data to create a concept map that emphasizes the strong interrelationship among deficiencies in water service delivery indicators and a constellation of resulting adverse life impacts faced by the urban poor (Fig 2 and Table 1). We also suggest ways in which these adverse life impacts may be measured in future studies using specific outcome indicators (Fig 2).

Most prior water-related studies in slums have presumed health, especially diarrheal illness, to be the sole outcome of interest [4–8, 10, 11], with a few exceptions. Studies of slums in Manila, Philippines [12]; Buenos Aires, Argentina [13]; Cochabamba, Bolivia [14]; and Mumbai, India [19] evaluated the impact of water service delivery failures on household income, expenditures, and psychological distress, respectively. Failure to evaluate non-diarrhea outcomes such as these may result in underestimation of the deprivation resulting from a poor water supply.

Viewing water service delivery in this manner also helps us to propose a definition of “household water poverty” as consisting of “deficiencies in one or more key water service delivery indicators that result in substantial negative health, economic, or social outcomes for the urban poor.”

This multidimensional definition of household water poverty is analogous to Amartya Sen’s definition of poverty more generally as deprivation in any of a variety of human capabilities (rather than deprivation in a single indicator such as income or wealth) [26]. Our definition differs somewhat from the “water poverty index” (WPI) described by Sullivan and colleagues due to our focus on evaluating water poverty at the household level [27, 28]. The WPI is geared more toward assessing water poverty at the national or community level, with indicators at the household level being somewhat more limited. Our definition incorporates a broader set of household-level indicators—quality, quantity, access, reliability, affordability, and equity—that are included in recent frameworks for monitoring water service delivery [23].

Our comparison of two groups in KB with different modes of water access—hose water recipients versus water fetchers—highlights the usefulness of a multidimensional definition of water poverty. Both groups suffer from “water poverty”—but for different reasons. Water fetchers face severe hardship with regard to water access, resulting in negative impacts on employment and income. In contrast, despite benefiting from relative ease of water access, hose water recipients suffer from poorer reliability and lower water quantity. Evaluating only one water service delivery indicator would provide an incomplete picture of water poverty for each group.

Water quantity is an especially important indicator as it maps onto multiple adverse life impacts and is independently associated with health outcomes (Fig 2) [17]. In our regression analysis, the price paid for water by a household is by far the strongest predictor of quantity of water used. A study of a slum in Manila similarly found that high water prices substantially limit the quantity of water consumed, despite every household having access to a tap within 100 meters [12]. These findings highlight the importance of ensuring that water is affordable, to enable slum dwellers to achieve basic human rights standards for water quantity (e.g., at least 50 LPCD).

Substantial inequity exists among households in KB with regard to water price and quantity. The high Gini coefficients suggest that some KB residents face extensive discrimination when obtaining water from informal water vendors. Because many slums are legally barred from accessing city water supplies, obtaining water from informal vendors is a common practice in slums globally [10, 13, 15, 29, 30]. While some urban planners suggest that city governments should partner with informal distributors to facilitate water delivery in slums [31], our findings encourage approaching such partnerships with caution, as they run the risk of further alienating marginalized groups in these communities.

How might application of a multidimensional framework help to reduce water poverty in slums? This framework has practical relevance for researchers, governments, and slum communities attempting to improve water service delivery in developing country cities.

For researchers, measurement of the non-health outcome indicators we propose in Fig 2 may be useful for better estimating the benefits of upgrading community water supplies in future quasi-experimental or randomized trials. Such data may help to provide better estimates of the cost-effectiveness and return on investment resulting from equitable water provision—data that may persuade governments to more rapidly upgrade water supplies. Because inadequate water access is one of the features that defines a slum based on the UN definition, better water metrics may also improve the accuracy of estimates of the slum population in India and globally [3].

For city governments, regular monitoring of multiple water service delivery indicators in slums will facilitate meeting international benchmarks for water provision, such as targets laid out in the post-2015 Sustainable Development Goals (SDGs) [32]. Currently, many city governments engage in minimal monitoring of water service delivery at the community or household level. For example, Mumbai’s government releases public reports on point-of-source bacterial contamination of the city water supply but collects minimal household-level data on other indicators [33]. The National Family Health Survey (NFHS)—India’s premier nationwide health survey—only measures the somewhat crude Millennium Development Goal (MDG) indicator of access to an “improved water supply” at the community level, a metric that has received substantial criticism [34–37].

For residents of slums, community-led measurement of water indicators may provide information that citizens can use to negotiate with governments to improve service delivery. The “right to research” is the idea that marginalized populations can collect strategic information to facilitate government accountability for basic service delivery [38, 39]. In Mumbai, slum dwellers’ federations have used household enumeration and mapping to prevent forced evictions and to enable community-managed resettlement [40, 41]. In India and Uganda, community-based monitoring of health services has been shown to increase healthcare utilization and dramatically improve child mortality [42, 43]. Similar community-driven initiatives focused on monitoring the water supply may empower communities to claim the human right to water from governments through negotiation and activism.

This mixed methods study of a Mumbai slum highlights the importance of assessing multiple water service indicators and identifies adverse impacts resulting from deficiencies in service delivery. In the emerging post-2015 development agenda, the widespread application of multidimensional monitoring and evaluation surveys in slums will be vital for accurately measuring the scale of water poverty and ultimately eliminating it.

## Supporting Information

S1 Appendix Tables A—C. Table A. Kaula Bandar’s demographic composition and key water indicators. Table B. Adverse life impacts of deficiencies in water service delivery in Kaula Bandar based on analysis of the qualitative data. Table C. Predictors of quantity of water used in the household in a multivariate ordinary least squares regression model.
(PDF)

S2 Appendix Discussion of methods for measuring the quantity of water used by households. (PDF)

S3 Appendix Study questionnaire and coding sheet. (PDF)
