# Supplementary material for: Multidimensional Measurement of Household Water Poverty in a Mumbai Slum: Looking Beyond Water Quality
Source: PLoS One. 2015 Jul 21;10(7):e0133241. doi: 10.1371/journal.pone.0133241 (PMC4511227; doi:10.1371/journal.pone.0133241)
Supplement: S1 Appendix — Table A. Kaula Bandar’s demographic composition and key water indicators. Table B. Adverse life impacts of deficiencies in water service delivery in Kaula Bandar based on analysis of the qualitative data. Table C. Predictors of quantity of water used in the household in a multivariate ordinary least squares regression model. (PDF) [file pone.0133241.s001.pdf]

S1 Appendix. Tables A-C

Table A. Kaula Bandar's demographic composition and key water indicators

	Prevalence in overall sample (N=521)<br><br>*N (%)*
**Demographics**	
Religion	
Muslim	255(48.9)
Hindu	241(46.3)
Christian, Buddhist	25(4.8)
Region of origin	
North Indian	246(47.2)
South Indian	164(31.5)
Maharashtrian	80(15.4)
Nepali	31(5.9)
Home ownership status	
Owns his/her living space	326(62.6)
Rents his/her living space	195(37.4)
Household monthly income (per capita)	
INR[a]<1000	94(18.4)
INR 1000-1499	152(29.7)
INR 1500-1999	116(22.7)
INR 2000-2499	51(10.0)
INR 2500+	99(19.3)
**Predominant location of defecation**	
Use of a community toilet	435(83.5)
Open defecation	86(16.5)
**Mode of water access**	
Residents who fetch water ("water fetchers")	179(34.4)
Residents who have water delivered via informal vendors ("hose water recipients")	339(65.1)
Residents who have taps in their homes	3(0.6)
**Water quantity**	
Average water quantity used in LPCD[(2)]<br>*Median (IQR)*	23.4(13.9,38.6)
Use of ≤20 LPCD of water	220(42.2)
Use of ≤50 LPCD of water	439(84.3)
**Price / water economics**	
Average water price in INR per 1000 liters<br>*Median (IQR)*	135.1(83.3,217.6)
Number of times the standard municipal water charge of INR 3.5 per 1000 liters of water<br>*Number of times the municipal rate*	38.6
Price of water	
INR <100 per 1000 liters	164(31.5)
INR 100-199 per 1000 liters	209(40.1)
INR 200+ per 1000 liters	148(28.4)
Monthly water spending as a percentage of monthly household income	mean=9.5%

0-4.9%	180(34.5)
5-9.9%	192(36.8)
10-19.9%	82(15.7)
20% or above	67(12.9)
**Frequency of water access**	
Number of times water was obtained in the last week	
1 time	148(28.5)
2-3 times	289(55.6)
4 or more times	83(16.0)
**Adverse impacts on work and school**	
Self-reported negative impact of getting water on work (e.g., late for work, missed work, or come back early from work)	196(37.6)
Self-reported negative impact of getting water on school[3] (e.g., late for school, missed school, or lost time studying)	91(26.6)

[1]INR=Indian rupees. [2]LPCD=liters per capita per day. [3]Out of a subset of 342 households with at least one child less than 14 years of age.

Table B. Adverse impacts of deficiencies in water service delivery in Kaula Bandar based on analysis of the qualitative data

**Adverse impact**	**Water service delivery failures causing the adverse impact**	**Representative quotations illustrating the adverse impacts from service delivery failures**
Economic and occupational impacts		
Opportunity costs due to time commitments involved in water fetching, which substantially limits employment options[1]	• Access (primary)<br><br>• Quantity (secondary)	"My boss called me today, but I didn't go to work because I had to fill water this evening. There is no water in my home."<br>- *20 year old Muslim man from Uttar Pradesh (a water fetcher)*<br><br>"[W]e spend three or four hours of the day just getting that water . . . It is extremely difficult for me to handle . . . In those three hours, we can't do any other work."<br>- *42 year old Muslim man from Uttar Pradesh (a water fetcher)*
Missed days of work due intermittent systemic failure of informal distribution system for prolonged periods due to government raids on motorized pumps used to extract water, resulting in men coming home early or staying home from work to fetch water[2]	• Reliability (primary)<br><br>• Quantity (secondary)	"Last week, I was in Goa for my job. My parents called me and told me to come back to Kaula Bandar because there was no water. So I left that job and I came back. I found that there was no water in this community."<br>- *19 year old Muslim man from Uttar Pradesh (a hose water recipient)*
Missed days of work due to shame over body odor due to inability to bathe	• Quantity	"I face similar problems. I work at a call center. Our call center rule is that you always have to look fresh. Sometimes I skipped work because there was no water to take a bath."<br>- *25 year old Muslim man from Uttar Pradesh*
Childcare and domestic work for women is compromised as they spend substantial time fetching water or waiting for water to arrive through the informal distribution system	• Reliability (primary)<br><br>• Access (primary)<br><br>• Quantity (secondary)	"We may not be watching over our children, but you can be sure we'll be watching for when the water comes in . . . Sometimes the water comes at 2 AM, 3 AM, 4 AM, but whenever it comes we have to be there waiting for it. It doesn't run on any timetable."<br>- *30 year old Hindu man from Tamil Nadu*

Water is sometimes the predominant expense in the household budget, cutting into money available for food and other necessities	• Price (primary)<br><br>• Quantity (secondary)	"We need money for everything. For water, we have to buy it every day. There is no electricity meter, so we pay money for electricity. I earn so little and I have so many expenses, so that causes tension. How can I manage everything? Every day we need to decide whether we want food or water."<br>- *30 year old Muslim woman from Uttar Pradesh*
Indebtedness sometimes results from loans being taken out to pay for water	• Price (primary)<br><br>• Quantity (secondary)	"We take out loans sometimes; just when we finish repaying those loans, we often need to take out another loan. I spend a lot of money on purchasing water . . . Everything is expensive, everything costs money."<br>- *55 year old Hindu woman from Tamil Nadu*
Educational impacts for children and youth		
Missed days or delayed arrival at school for children, due to time spent fetching water[a]	• Access (primary)<br><br>• Quantity (secondary)	"[W]e go to Sewri, Reay Road, or Cotton Green Station to get water and bring it back from there . . . My older and younger sons [ages 14 and 16 years old] both go and get water there every day."<br>- *50 year old Muslim man from Uttar Pradesh*<br><br>"We get very little water here. Yesterday, this small child [her 10 year old son] went to Reay Road to bring water back to our home . . . [We] have to travel a long distance to purchase water."<br>- *30 year old Muslim woman from Uttar Pradesh*<br><br>"How can she get an education if there is no water in our area! People are always sending their children to bring water. Because they fill water for their home, they never go to school."<br>- *40 year old Hindu man from Tamil Nadu*
Missed days of school during periods when the informal distribution system fails, which results in children spending the entire day fetching water[b]	• Reliability (primary)<br><br>• Quantity (secondary)	"Sometimes my children are late getting to school because they have to wait for water to be delivered, and then the teacher punishes them."<br>- *40 year old Hindu woman from Maharashtra*
Missed days of school due to shame over body odor due to inability to bathe	• Quantity (primary)<br><br>• Reliability (secondary)<br><br>• Access (secondary)	"Woman 1: We have small children in my home. When they go to school without taking a bath, they face problems there. Woman 2: Teachers blame us for sending our children to school without giving them a bath. They don't understand the problems we face here."<br>- *60 year old Muslim woman from Uttar Pradesh and 33 year old woman from Tamil Nadu in a women's focus group discussion*<br><br>"We never take a bath, because we don't have enough water. Sometimes I missed college because I don't like to go to class without taking a bath. People who live here often don't take a bath for two to three days. For the last week, we only washed our faces; we didn't take a bath. We wash our faces and go to work. When we come back, we fill water for our homes."

		- *19 year old Muslim man from Uttar Pradesh*<br><br>"As I told you, I don't get enough water to clean my children. Every day I clean each of them with only four glasses of water each. For my mentally disabled child, I use more water because I am concerned about his health. I don't bathe every day . . . If I don't clean my children every day then how could they go to school? The other children at school tease them, but what can I do? It's a serious problem. Most of the time my body smells dirty, but I can't clean myself. Then I get frustrated. My children want to bathe in more water, but it's impossible. So they are also irritated."<br>- *35 year old Hindu woman from Uttar Pradesh*
Studying and homework time is compromised by water collection duties for school-age children[a]	• Access (primary)<br><br>• Quantity (secondary)	"My children have to fill up the water every day; even if they have to study for an exam they still go to get the water."<br>- *40 year old Hindu woman from Maharashtra*<br><br>"Because [my children] are always going to fetch water, they can't concentrate on their studies. My child failed his last three assignments in Math, English, and Marathi, because he was always going back and forth to get water. Because of our water crisis, our children are losing their education. One of my sons had an economics paper to do, but he spent all day filling water, and that's why he failed on that paper. If they go outside of the community to fill water, then our children end up spending one to one and a half hours just for one trip to fill water."<br>- *40 year old Muslim woman from Uttar Pradesh*
Health impacts		
Post-source contamination of water is a concern due to the challenging process of navigating water containers from the point-of-source taps back to their homes[a]	• Quality (primary)<br><br>• Access (secondary)	"[W]e spend a lot of time rolling drums up and down the road to get water. Since we have to roll our water drums by the overflowing garbage bins on the road, I'm always afraid that our water will get contaminated by trash."<br>- *30 year old Hindu woman from Tamil Nadu*
Post-source contamination of water is a concern due to very prolonged periods of storage of water in unsafe containers prior to consumption[b]	• Quality (primary)<br><br>• Reliability (secondary)	"People who live here, they often don't get drinking water for more than a week. Sometimes bugs grow in our drinking water containers, and that's why people get sick."<br>- *26 year old Hindu man from Maharashtra*
"Foul" smell or unappealing appearance of water makes consumption unpleasant and leads to the perception of contamination	• Quality	"Sometimes our water has a foul smell. But we have to store water 2-3 days and then have to drink that water. We know that water is dirty, but we still use it because we don't get fresh water."<br>- *40 year old Muslim woman from Uttar Pradesh*<br><br>"The water we get is of poor quality. It looks like there is dirt in it sometimes. We boil it, but my girls still get sick all the time. So if we just got water, everything would become much easier."<br>- *30 year old Hindu man from Tamil Nadu*

		"We get clean water, but only once or twice a week, not every day. I have my own water motor, but sometimes sea water comes out of that motor and sometimes there are little bugs and mud in that water."<br>- *56 year old Hindu man from Tamil Nadu*<br><br>"There were only four times where we got water during this entire last month. So sometimes we have to store water for as much as 10 days. When we do this, we find all kinds of bugs in the water. But we don't have any choice, so we drink that water."<br>- *28 year old Hindu woman from Tamil Nadu*
Body hygiene, hygiene of cooking and eating utensils, cleanliness of clothes, and cleanliness of the home is difficult to maintain due to lack of water	• Quantity (primary)<br><br>• Reliability (secondary)<br><br>• Access (secondary)	"We spend lots of money on water but we still don't get enough. If my children take a bath then there is not enough water for me to bathe."<br>- *40 year old Hindu woman from Uttar Pradesh*<br><br>"Wife: [The water situation] is unbearably difficult. Sometimes we've gone ten days without bathing.<br>Husband: Even now I go two or three days without bathing. Sometimes we don't have water to drink."<br>- *48 year old Hindu husband and wife from Tamil Nadu*<br><br>"Sometimes we don't get water for a week. I can't clean my home because I don't have enough water, so my home stinks. I don't like it, but I have no choice. There is not enough water to bathe my children. If guests come to my home then I am embarrassed. I know how important it is to clean, but I can't clean my clothes and utensils properly. And then we get sick. I know all these things, but I don't know how to deal with this problem."<br>- *35 year old Hindu woman from Maharashtra*
Toilet seats in community block toilet structures rapidly deteriorate due to lack of sufficient water for maintenance	• Quantity (primary)<br><br>• Reliability (secondary)<br><br>• Access (secondary)	"PUKAR Researcher: But the Sulabh [pay-per-use block] toilets are clean, right?<br>Woman 1: It's clean but the people here make it dirty.<br>Woman 2: The main problem is that the toilet seats are not cleaned every day because there is no proper water supply."<br>- *24 year old Hindu woman from Uttar Pradesh and 45 year old Hindu woman from Maharashtra in a focus group discussion*
Hygiene in public spaces is poor due to lack of water for cleaning	• Quantity (primary)<br><br>• Reliability (secondary)<br><br>• Access (secondary)	"Come with us and see the condition of our lanes. We try to keep our lanes clean, but our children come from the outside and make them dirty. The whole day I clean my lane, but we don't get enough water to clean the lanes well."<br>- *30 year old Muslim woman from Uttar Pradesh*

Drinking water availability is compromised on rare occasions when scarcity is severe, leading to risk of dehydration	• Quantity (primary)<br><br>• Reliability (secondary)<br><br>• Access (secondary)	"Suppose water doesn't come for ten days. After five days, we begin to run out of water, and I can't complete any of my housework . . . In the end, we always have to set aside some water to make sure that we have enough to drink. Even if we aren't washing the clothes or the dishes or cooking, we have to make sure we have enough to drink."<br>- *30 year old Hindu woman from Tamil Nadu*<br><br>"Even now I go two or three days without bathing. Sometimes we don't have water to drink."<br>- *48 year old Hindu man from Tamil Nadu*<br><br>"We go through all that struggle, get the water, bring it home, and then the next day we can have a glass water. Otherwise we have to go to the next house and beg them for water. If we take one vessel full of water from the house next door, we can be sure that they will ask for that same vessel full of water back the next day."<br>- *30 year old Hindu man from Tamil Nadu*
Impacts on quality of life		
Physical strain from water fetching, the toll of which is especially evident on the elderly, women, and children[a]	• Access (primary)<br><br>• Quantity (secondary)	"The biggest problem with that is trying to bring barrels of water back to my home. If we have to get water from outside, getting the barrel through the lane to get the water and then bring it back becomes an impossible task because the lanes are so small. We have to roll it foot-by-foot and push other people's things and ladders out of the way to get it through the lane. Just to bring one barrel from the road all the way through the lane to our house takes an hour."<br>- *30 year old Hindu man from Tamil Nadu*<br><br>"We have to push the water drum all the way from our home to beyond the police station [outside of Kaula Bandar] where the taps are. Now that we're getting old, it is difficult . . . There's never enough room to bring the drum back through the lane. Look at how small our lane is! So most of the time we don't take the big drums to get water, and we just carry water in small containers on our heads. We take vessels like these [points to 15-liter metal vessels]."<br>- *48 year old Hindu woman from Tamil Nadu (a water fetcher)*<br><br>"[W]e have to bring water back to our homes in those big blue drums. It's easy to roll those drums back and forth down the road. But it's incredibly difficult to maneuver those drums through the lanes to get them back to our homes. Since the drums don't fit through the lanes easily, we end up having to carry water back to our home in smaller metal containers."<br>- *30 year old Hindu woman from Tamil Nadu*
Loss of sleep due to substantial time commitments involved in water fetching[a]	• Access (primary)<br><br>• Quantity (secondary)	"Sometimes we go to all the way to Road or Dockyard Road to get our water. Last summer, we were staying up all night just filling water. No one delivers water to my home."<br>- *42 year old Muslim man from Uttar Pradesh*
Frustration due to the unpredictable nature of water flow through the informal distribution	• Reliability (primary)	"Sometimes the water comes at 2 AM, 3 AM, or 4 AM, but whenever it comes we have to be there waiting for it. It doesn't run on any timetable."<br>- *30 year old Hindu man from Tamil Nadu*

system[2]	• Quantity (secondary)	
Frustration emerges due to the unpredictability of when water will arrive in any given week[b]	• Reliability (primary)<br><br>• Quantity (secondary)	"We wait and wait, and then sometimes [water] will come once in a week. So for one whole week we end up using the same water."<br>- *30 year old Hindu man from Tamil Nadu*
Frustration on the part of women over inability to complete basic domestic work, including washing clothes, cleaning the home, and bathing children due to lack of water	• Quantity (primary)<br><br>• Reliability (secondary)<br><br>• Access (secondary)	"The hardest part for me is that I just can't get my housework done. The morning is very stressful, because I have to get the children to school, and everyone has to take a bath before they leave. That's hard if we don't have enough water. Sometimes we can't wash our clothes as often as we'd like. So I feel frustrated that I just can't get any work done. Sometimes some of us won't leave the house because we are unable to take a bath."<br>- *32 year old Christian woman from Tamil Nadu*<br><br>"Sometimes we don't get water for a week. I can't clean my home because I don't have enough water, so my home stinks. I don't like it, but I have no choice. There is not enough water to bathe my children. If guests come to my home then I am embarrassed. I know how important it is to clean, but I can't clean my clothes and utensils properly. And then we get sick. I know all these things, but I don't know how to deal with this problem."<br>- *35 year old Hindu woman from Maharashtra*<br><br>"Suppose water doesn't come for ten days. After five days, we begin to run out of water, and I can't complete any of my housework. During those times, my main preoccupation is wondering when water will come again. All of our clothes accumulate in the house, because we can't wash them. If we had water, we could actually complete our daily household chores. During times when water doesn't come, I have a lot of tension. At some point, even cooking food becomes difficult. In the end, we always have to set aside some water to make sure that we have enough to drink. Even if we aren't washing the clothes or the dishes or cooking, we have to make sure we have enough to drink . . . Water usually comes to us again after one week. If water doesn't come after more than a week, it's unbearable."<br>- *30 year old Hindu woman from Tamil Nadu*<br><br>"The only problem I have with housework is getting enough water to actually do my household. That's a difficult job. If we just received regular water here, everyone's troubles would be much better. The housework only gets done if I have water."<br>- *30 year old Hindu woman from Tamil Nadu*
Feelings of shame among members of severely water poor households, as they sometimes have to beg neighbors for water[b]	• Quantity (primary)<br><br>• Reliability (secondary)	"We go through all that struggle, get the water, bring it home, and then the next day we can have a glass water. Otherwise we have to go to the next house and beg them for water. If we take one vessel full of water from the house next door, we can be sure that they will ask for that same vessel full of water back the next day."<br>- *30 year old Hindu man from Tamil Nadu*

	• Access (secondary)	
Feelings of shame develop over body odor and poor hygiene, especially at work or at school	• Quantity (primary)<br><br>• Reliability (secondary)<br><br>• Access (secondary)	**“**As I told you, I don’t get enough water to clean my children. Every day I clean each of them with only four glasses of water each . . . If I don’t clean my children every day then how could they go to school? The other children at school tease them, but what can I do?”<br>- *35 year old Hindu woman from Maharashtra*<br><br>“Sometimes I missed college because I don’t like to go to class without taking a bath. People who live here often don’t take a bath for two to three days. For the last week, we only washed our faces; we didn’t take a bath. We wash our faces and go to work. When we come back, we fill water for our homes.”<br>- *19 year old Muslim man from Uttar Pradesh*
Impacts on social relationships, community cohesion, and feeling of political inclusion		
Relatives from rural areas are unable to visit due to lack of water, resulting in estranged relationships	• Quantity (primary)<br><br>• Reliability (secondary)<br><br>• Access (secondary)	**“**Suppose our relatives come from our native villages in Tamil Nadu. In their homes, they have their own bathrooms and they have many amenities. But during the ten days they spend here, they are shocked at how hard it is to go to the bathroom and get water. So then they leave early. And they always say to me, ‘Things are so easy where we live, it’s easy for us to get water.’ They usually leave within four days.”<br>- *48 year old Hindu woman from Tamil Nadu*<br><br>“Sometimes we don’t get water for a week. I can’t clean my home because I don’t have enough water, so my home stinks . . . If guests come to my home then I am embarrassed.”<br>- *35 year old Hindu woman from Maharashtra*
Community events such as weddings and festivals are limited due to lack of water, and the substantial expenses required to hire water tankers to ensure adequate water for these events	• Quantity (primary)<br><br>• Reliability (secondary)<br><br>• Access (secondary)	“Ramzan is a big festival for us. But during that festival, sometimes we don’t get water. So we can’t keep ourselves clean. Water is a big problem.”<br>- *40 year old Muslim woman from Uttar Pradesh*<br><br>“Last time I had a function [ceremony or gathering] in my home, we didn’t get water that day. So just imagine the problems we faced at that time. Because of that, we didn’t take a bath that day.”<br>- *30 year old Muslim woman from Uttar Pradesh*
Social conflict when navigation of water drums through narrow slum lanes impinges on other peoples’ property and common space[a]	• Access (primary)<br><br>• Quantity (secondary)	“It’s especially a problem when we are trying to bring water back to our homes. There’s not enough room to maneuver the blue drums through the lanes. Inevitably, we accidentally knock someone’s things down in the lane and get into an argument with them. If one person is standing, then no one else can walk down the lane.”<br>- *30 year old Hindu man from Tamil Nadu*

Feelings of resentment, bitterness, and distrust emerge against the water vendors, who also live in the community, and who are perceived of as being unfair towards individuals who get water from them[b]	• Equity (primary)<br><br>• Price (secondary)<br><br>• Reliability (secondary)	"Every month, we pay [the water vendors] 500 rupees for water, but there is no guarantee that we will get regular water. Some days, we don't get any water [from the water vendors], so we go outside of Kaula Bandar to fetch water. We end up spending 50 rupees extra that day, on top of the trouble of bringing water from outside of the community. But the water vendors still collect their monthly charges, whether they actually deliver water or not. They will never give us a concession if water doesn't actually come. Sometimes they don't provide any water for 10 to 15 days."<br>- *30 year old Muslim woman from Uttar Pradesh*<br><br>"We go to the corner of our lane to fill our containers with water. If you fill your container and leave it there on the corner briefly, because there is no one to bring it back home, then the water vendor throws your containers away and says, 'If you don't want to take them then why are you wasting my time?'"<br>- *40 year old Hindu woman from Uttar Pradesh*<br><br>"Every day we have to pay the water mafia [i.e., the water vendors] for water. But if the government would provide us with water, we are ready to pay them . . . [The water vendors] demand whatever fees they want for water, and because we have no other option, we have to pay those fees. For 400 rupees we only get water for 15 days a month at most . . . If we stopped purchasing water from the water vendors, then where would we get water? . . . They tell us if we can't pay the money, then we can get water from someone else."<br>- *40 year old Hindu woman from Maharashtra*<br><br>"The water mafia runs its own system here. The government doesn't help us. The politicians tell us, 'We will help you,' but then they do nothing . . . The water mafia charges whatever price they want. They only give water once a week. We can't speak out against them, because they are thugs."<br>- *38 year old Christian man from Kerala*
Police raids on water vendors' motorized pumps result in a sense of collective punishment and social exclusion	• Equity (primary)<br><br>• Reliability (secondary)<br><br>• Access (secondary)<br><br>• Price (secondary)	"Now and then the [Mumbai Port Trust] shut[s] off all the water to everyone here. At most we get twice a week, and on top of that they are now shutting off the water supply completely at times. How many people have suffered because of this?"<br>- *30 year old Hindu man from Tamil Nadu*<br><br>"Sometimes the government officer come and raid the water motors. I have to deal with that stress also. I've lost four motors that way. The [Mumbai Port Trust] officers comes here and take away all the motors. When they take them, we never get them back. Each time it happens, I purchase a new motor and spend a lot of money on it. Sometimes the police cut our water supply. Other local residents would never do that to our water supply."<br>- *56 year old Hindu man from Tamil Nadu*<br><br>"The [Mumbai Port Trust] officials come and take away the water vendors' motors sometimes. When this happens, we face major problems. The water sellers are usually paying the [Mumbai Port Trust] officials huge amounts of money in bribes to keep their motors running, so they don't get raided. We end up paying all of those costs in the end! Our monthly expenditure on water is very high."<br>- *36 year old Buddhist man from Tamil Nadu*

		“We purchase water from a water vendor, and he delivers the water through a hose to our home. But sometimes the [Mumbai Port Trust] raids his motor. Then for 15-20 days he can’t distribute water. So we have to go outside of Kaula Bandar to fetch water . . . For the last 18 years we’ve had this problem . . . When the [Mumbai Port Trust] takes the water motors, then we have big problems. About every two or three months the [Mumbai Port Trust] comes and takes the motors.”<br>- *31 year old Muslim man from Uttar Pradesh*<br><br>“We have this water crisis primarily because we are on [Mumbai Port Trust] land. I feel like they are stopping the water because they eventually want us to leave this land.”<br>- *32 year old Christian woman from Tamil Nadu*
Bribes extorted by the police for attempting to access alternative water sources compromises the relationships between residents and the police	• Equity (primary)<br><br>• Price (secondary)	“Last week, I was in Goa for my job. My parents called me and told me to come back to Kaula Bandar because there was no water. So I left that job and I came back. I found that there was no water in this community. One of my friends lives near Victoria Terminus. Near his home, there is a well. I told him, ‘I am sending a water tanker; please fill that. Then I will distribute that water in my community.’ We did that two times. By the third time, the police stopped me. They asked me, ‘Where did you get this water?’ I told them, ‘I got this water at VT.’ They fined me 1800 rupees. They told me that it is illegal to bring water into the community. If you want to bring water, then you have to take permission from them. Then I stopped bringing water to the community, because I had to pay out of my own pocket.”<br>- *19 year old Muslim man from Uttar Pradesh*
Cynicism about the democratic process emerges among residents due to the repeated failure of politicians and government officials to improve the water situation in Kaula Bandar	• Equity	“Big political leaders come [to Kaula Bandar] and promise that they will bring water here, but, after all these years, we still have to walk so far to fetch water.”<br>- *45 year old Hindu woman from Maharashtra*<br><br>“I’ve been living here for the last 33 years. Some people have lived here for more than 40 years. And yet we still don’t have a water supply. Like me, many people in Kaula Bandar work for the BMC [the city government], and yet, the BMC won’t provide water to its own workers.”<br>- *34 year old Hindu man from Tamil Nadu*<br><br>“In the past, a politician came, placed a water pipe around the time of an election. After the election, the politician just left the work as it was without completing it. That pipe is still sitting in the ground, useless. He won the election and then just forgot about us. Even now they’re digging up the road, placing a new pipe for the next election.”<br>- *30 year old Hindu man from Tamil Nadu*<br><br>“Every day a human being needs water. But we never get as much water as we need. We have tried so many different strategies to get better water access in Kaula Bandar. We have met with government officers, but still nothing has happened. We now spend so much money on water that we are considering moving out of Kaula Bandar.”<br>- *25 year old Hindu man from Tamil Nadu*<br><br>“The water mafia runs its own system here. The government doesn’t help us. The politicians tell us, ‘We will help you,’ but then they do nothing . . . The water mafia charges whatever price they want. They only give water once a week. We

	can't speak out against them, because they are thugs."<br>- *38 year old Christian man from Kerala*<br><br>"During election time, politicians come and promise us that if we vote for them they will bring a water supply and build toilets here after the election. But no one actually does it. They only come here for the votes."<br>- *45 year old Muslim man from Maharashtra*<br><br>"In the last three years the water situation in Kaula Bandar has gotten worse compared to the past. We put in applications with the police station and the [Mumbai Port Trust] office, and they promise us we will get a water supply, but we still never have. Politicians always make promises but never do anything."<br>- *31 year old Muslim woman from Maharashtra*

[a]Primarily affects water sellers; [b]Primarily affects hose water recipients

Table C. Predictors of quantity of water used in a multivariate ordinary least squares regression model

**Risk factor**	**Univariate Findings**<br><br>*β-coefficient (p-value)*	**Multivariate findings** (N=508, R^2=0.5187)<br><br>*β-coefficient (CI)*	**p-value**
**Demographics**			
Number of people in the household *(per each one person increase)*	-2.34 (<0.001)	-2.64 (-3.33, -1.96)	<0.001*
Religion			
Hindu	-	-	-
Muslim	2.24 (0.357)	-0.69 (-4.54, 3.16)	0.724
Christian or Buddhist	10.59 (0.061)	3.60 (-4.67, 11.87)	0.393
Region of origin			
North Indian	-	-	-
South Indian	7.29 (0.007)	5.08 (0.47, 9.69)	0.031*
Maharashtrian	-1.16 (0.736)	-1.03 (-6.00, 3.95)	0.686
Nepali, other	2.17 (0.671)	0.57 (-6.42, 7.56)	0.873
Rents his/her living space	-9.40 (<0.001)	-4.17 (-7.94, -0.41)	0.030*
Household monthly income per capita *(per each INR[a] 100 increase)*	0.37 (<0.001)	0.28 (0.14, 0.42)	<0.001*
**Water-related variables**			
Cost of water in INR per 1000 liters of water	-0.55 (<0.001)	-0.46 (-0.56, -0.36)	<0.001*
Water fetcher *(as compared to being a hose water recipient)*	15.58 (<0.001)	5.64 (1.92, 9.36)	0.003*
Frequency of obtaining water in the last week *(per each increase in number of times water was obtained)*	9.26 (<0.001)	8.04 (6.92, 9.13)	<0.001*
Model constant	-	26.36 (19.22, 33.5)	<0.001*

[a]INR=Indian rupees
