# Supplementary material for: Multidimensional Measurement of Household Water Poverty in a Mumbai Slum: Looking Beyond Water Quality
Source: PLoS One. 2015 Jul 21;10(7):e0133241. doi: 10.1371/journal.pone.0133241 (PMC4511227; doi:10.1371/journal.pone.0133241)
Supplement: S2 Appendix — (PDF) [file pone.0133241.s002.pdf]

S2 Appendix. Discussion of methods for measuring the quantity of water used by households.

In a recent meta-analysis, use of an inadequate quantity of water was found to be independently associated with poor health outcomes (1). Use of an inadequate quantity of water results in the deterioration of hygiene of the hands, body, clothes, and eating utensils, which may promote diarrheal, parasitic, and skin diseases. In addition, quantity is an indicator that may reflect deficiencies in other aspects of water service delivery (e.g., water timings, frequency, waiting times at taps, and distance from taps). As such, accurately measuring water quantity used by households should be a priority for city governments.

Despite the importance of this indicator, relatively few studies of slums have evaluated water quantity (2-7). The paucity of research on quantity may be due to difficulties in measuring water use in slums, where water meters are often absent and hundreds of people may use a single tap. Also, unlike water quality, quantity cannot be assessed using simple lab tests.

We conducted a literature review and identified ten studies that measured the quantity of water used by poor households in developing countries—where water meters are presumably absent (2-11). We found that a variety of often ad hoc methods were used, none of which have been critically evaluated for their accuracy. These include the following:

(1) Direct observation of households to assess water use. Such observation was often limited to 12 hours at a time and potentially reflective of a transitory Hawthorne effect, which refers to a person's usual behavior being modified due to knowledge that he or she is being directly observed (4, 5, 8).
(2) Water usage "interviews" (10).
(3) Provision of graduated pitchers to research subjects to facilitate quantification of water used, which is also potentially subject to the Hawthorne effect (4).
(4) Container enumeration methods, similar to the one used in our study of KB (2, 6, 7, 9, 11).

For our research in KB, we use a container enumeration method in which we individually assess the volume that each household water container can hold and the number of times each container was filled with water in the previous seven days. We believe that this approach has excellent face validity. We use a one-week recall period because water collection in KB is usually intermittent, arriving every other day at best. Evaluating water use over one week therefore likely provides a better estimate of average water use than does assessing water use over a 24-hour recall period. Our container enumeration method also has the benefit of being relatively easy to implement, since a trained researcher can perform the assessment in about five minutes.

In a separate analysis, we evaluated the "construct validity" of the container enumeration method for identifying water-poor subpopulations within KB (12). "Construct validity" is the extent to which a test measures a given construct, based on pre-specified observation-based hypotheses about that construct. Using the qualitative data, we developed hypotheses regarding the likelihood that specific sub-populations in KB suffer from use of an inadequate quantity of water (12). The predictors of use of an inadequate quantity of water in our study are largely concordant with our pre-specified hypotheses. Therefore, we believe that our container enumeration method may have utility for identifying sub-populations who consume an inadequate quantity of water (12).

While these findings are encouraging, our container enumeration method also has potential shortcomings. Households may refill some of their containers with water before they are

completely empty. If this is the case, our method may slightly overestimate water quantity consumed. If anything, this would underestimate (rather than exaggerate) the proportion of households that suffer from access to an inadequate quantity of water. Our method may also have inaccuracies secondary to recall limitations. Establishing the validity of our method by comparing it to an alternative (but more time-consuming) assessment of water quantity using daily measurement is a goal for future research.

References

1. Stelmach RD, Clasen T. Household water quantity and health: a systematic review. Int J Environ Res Public Health. 2015;12(6):5954-74.
2. Subbaraman R, Shitole S, Shitole T, Sawant K, O'Brien J, Bloom DE, et al. The social ecology of water in a Mumbai slum: failures in water quality, quantity, and reliability. BMC Public Health. 2013;13:173.
3. Oswald WE, Hunter GC, Lescano AG, Cabrera L, Leontsini E, Pan WK, et al. Direct observation of hygiene in a Peruvian shantytown: not enough handwashing and too little water. Trop Med Int Health. 2008;13(11):1421-8.
4. Gilman R, Grace S, Ventura G, Campos M, Spira W, Diaz F. Water cost and availability: key determinants of family hygiene in a Peruvian shantytown. American Journal of Public Health. 1993;83:1554-8.
5. Cairncross S, Cliff JL. Water use and health in Mueda, Mozambique. Trans R Soc Trop Med Hyg. 1987;81(1):51-4.
6. Aiga H, Arai Y, Marui E, Umenai T. Impact of improvement of water supply on reduction of diarrheal incidence in a squatter area of Manila. Environ Health Prev Med. 1999;4(3):111-6.
7. Aiga H, Umenai T. Impact of improvement of water supply on household economy in a squatter area of Manila. Soc Sci Med. 2002;55(4):627-41.
8. Bailey R, Downes B, Downes R, Mabey D. Trachoma and water use; a case control study in a Gambian village. Trans R Soc Trop Med Hyg. 1991;85(6):824-8.
9. West S, Lynch M, Turner V, Munoz B, Rapoza P, Mmbaga BB, et al. Water availability and trachoma. Bull World Health Organ. 1989;67(1):71-5.
10. Verweij PE, van Egmond M, Bac DJ, van der Schroeff JG, Mouton RP. Hygiene, skin infections and types of water supply in Venda, South Africa. Trans R Soc Trop Med Hyg. 1991;85(5):681-4.
11. Whittington D, Mu X. Calculating the value of time spent collecting water: some estimates for Ukunda, Kenya. World Development. 1990;18(2):269-80.
12. Subbaraman R, Nolan L, Sawant K, Shitole T, Shitole S, Nanarkar M, et al. The construct validity of a novel method for quantifying water consumption in slum settlements in Mumbai, India. UNC Water and Health Conference; 2014 Oct 13-17; Chapel Hill, NC, U.S.A.; 2014. Available at: http://whconference.unc.edu/files/2014/10/subbaraman.pdf
