# Supplementary material for: Multidimensional Measurement of Household Water Poverty in a Mumbai Slum: Looking Beyond Water Quality
Source: PLoS One. 2015 Jul 21;10(7):e0133241. doi: 10.1371/journal.pone.0133241 (PMC4511227; doi:10.1371/journal.pone.0133241)
Supplement: S3 Appendix — (PDF) [file pone.0133241.s003.pdf]

S3 Appendix. Water Poverty and Mental Health Study Questionnaire and Coding Interpretation Sheet

Partners for Urban Knowledge, Action, and Research (PUKAR)

Version Date: January 11, 2012

Used in the following published studies:

Subbaraman R, Nolan L, Sawant K, Shitole S, Shitole T, Nanarkar M, Patil-Deshmukh A, Bloom DE. Measuring water poverty in a Mumbai slum: looking beyond quality. *PLOS One* 2015.

Subbaraman R, Nolan L, Shitole T, Sawant K, Shitole S, Sood K, Nanarkar M, Ghannam J, Betancourt TS, Bloom DE, Patil-Deshmukh A. The psychological toll of slum living in Mumbai, India: a mixed methods study. *Social Science and Medicine* 2014; 119:155-169. (PMID 25189736)

STUDY QUESTIONNAIRE

Section 1: Observation

Date of Survey ___________________

Interviewer's Name ___________________

Name of respondent ___________________

Household Code ___________________

Observation:

The next few questions are to be assessed by the researcher (not to be asked to the respondent):

1. Is this home:

☐ kutcha (only wood, mud, or tarp)

☐ semi-pucca (wood and metal, maybe some cement)

☐ pucca (only made of cement and durable materials)

2. Is this home within 20 meters of a large solid waste dump?

☐ Yes

☐ No

3. Is this home directly next to or on the ocean?

☐ Yes

☐ No

4. Is this home directly *on* the big road?

☐ Yes

☐ No

5. Is this home in a very narrow lane?

☐ Yes

☐ No

Section 2: Demographic and background information

We will first ask you a few questions to get some basic information about you and your household.

1. Gender:

☐ Male

☐ Female

☐ Transgender

2. Age ____________ years

3. Religion:

☐ Hindu

☐ Muslim

☐ Christian

☐ Buddhist

☐ Other ____________

4. Marital status:

☐ Never married

☐ Currently married

☐ Divorced

☐ Husband or wife left me

☐ Widowed (husband or wife has died)

5. What is the region of India in which your village is located (clarify with mother tongue if need be)?

☐ North Indian (UP or Bihar)

☐ South Indian (Tamilian)

☐ Maharashtrian

☐ Bengali

☐ Other ______________

6. Have you faced problems because of inability to speak, read, or write a local language?

☐ Yes

☐ No

7. What is the highest level of education that you have *completed* in a regular school?

________________ (highest standard completed)

8. What is the monthly income of your household?

☐ <3000 rupees

☐ 3000-3999 rupees

☐ 4000-4999 rupees

☐ 5000-5999 rupees

☐ 6000-6999 rupees

☐ 7000-7999 rupees

☐ 8000-8999 rupees

☐ 9000-9999 rupees

☐ 10,000-10,999 rupees

☐ 11,000-11,999 rupees

☐ >12,000 rupees (if so, write income ________)

☐ I don’t know

Section 3: General Health Questionnaire

FOR THE 12 QUESTIONS CONTAINED IN THE GHQ-12, PLEASE CONTACT THE AUTHORS OF THE GHQ, AS THIS IS COPYRIGHTED MATERIAL.

Do you believe that sadness or tension are illnesses?
□ Yes
□ No

Do you believe this illness can be treated by a doctor?
□ Yes
□ No

Section 4: WHO Disability Assessment Schedule 2.0

Health problems or tension can have an impact on the day-to-day activities you are able to do. We will now ask you some questions to assess difficulties you may have doing your activities. By difficulty in completing a task, we mean any increased effort, discomfort or pain, slowness, or change in the way that you do the activity.

In the past 30 days, how much difficulty did you have in:		None	Mild	Moderate	Severe	Extreme or cannot do
S1	Standing for long periods such as 30 minutes?	1	2	3	4	5
S2	Taking care of your household responsibilities?	1	2	3	4	5
S3	Learning a new task, for example, learning how to get to a new place?	1	2	3	4	5
S4	How much of a problem did you have joining in community activities (for example, festivities, religious or other activities) in the same way as anyone else can?	1	2	3	4	5
S5	How much have you been emotionally affected by your health problems?	1	2	3	4	5

In the past 30 days, how much difficulty did you have in:		None	Mild	Moderate	Severe	Extreme or cannot do
S6	Concentrating on doing something for ten minutes?	1	2	3	4	5
S7	Walking a long distance such as a kilometre [or equivalent]?	1	2	3	4	5
S8	Washing your whole body?	1	2	3	4	5
S9	Getting dressed?	1	2	3	4	5
S10	Dealing with people you do not know?	1	2	3	4	5
S11	Maintaining a friendship?	1	2	3	4	5
S12	Your day-to-day work/school?	1	2	3	4	5

H1	Overall, in the past 30 days, how many days were these difficulties present?	***Record number of days*** ____
H2	In the past 30 days, for how many days were you totally unable to carry out your usual activities or work because of any health condition?	***Record number of days*** ____
H3	In the past 30 days, not counting the days that you were totally unable, for how many days did you cut back or reduce your usual activities or work because of any health condition?	***Record number of days*** ____

Do you feel that you have any of the following problems that are permanent and severe enough to restrict your daily activities?
Trouble seeing (with eyeglasses, if you wear any) □ Yes □ No
Trouble hearing □ Yes □ No
Any difficulty with speech (i.e., speech impairment) □ Yes □ No
Paralysis of any part of the body (entire body, both legs, or one side of the body) □ Yes □ No
Loss of a leg, arm, hand, foot, or thumb □ Yes □ No

Section 5: Slum Adversity Questionnaire

Finally, we would like to ask you some questions about problems you may face from living in Kaula Bandar.

Overcrowding

1. How many home spaces in Kaula Bandar does your family occupy? ___________

2. How many total people are in your household? _____________

3. How many children (<14 years of age) live in your household? ___________

4. In the last three months, have you or any of your family members ever have to sleep outside (or in another person's home) because there is not enough space in your own home?

☐ Yes

☐ No

5. In the last three months, have you or any of your family members had to sleep sitting up in your home because of lack of space?

☐ Yes

☐ No

6. Do you have any conflicts with any neighbors in the next closest homes in your lane?

☐ Yes

☐ No

Insecure residential status

1. Is this house:

☐ Rented

☐ Owned by you or your family

2. Does your household have a ration card?

☐ Yes → Go to 2b, 2c, and 2d

☐ No → Go to 2a

2a. If no, have you faced tension from trying to get a ration card?

☐ Yes

☐ No

If yes, do you face tension from any of the following problems:
2b. Your name or a family member’s name is missing from the card

☐ Yes

☐ No

2c. Your name or a family member’s name is misspelled and this causes problems:

☐ Yes

☐ No

2d. Your household doesn’t get enough kerosene or grain from the ration card for your needs:

☐ Yes

☐ No

3. Do you have a PAN card?

☐ Yes

☐ No

4. Have you been threatened with eviction in the last year?

☐ Yes

☐ No

5. In the last five years, have you been evicted from a home in a city slum *by the owner of the home?*

☐ Yes → Go to 5a

☐ No

5a. If yes, how many times have you been evicted by an owner?_______

6. In the last five years, have you had your home demolished *by the BPT, BMC, or any company*?

☐ Yes → Go to 6a

☐ No

6a. If yes, how many times has this happened? ___________

Poor structural quality of housing
1. Has your home ever burned down due to a fire?

☐ Yes

☐ No

2. Have you ever experienced small fires in your lane or home that caused you anxiety?

☐ Yes

☐ No

3. Does your home flood significantly with water at anytime (because of monsoon or backup through a drainage line)?

☐ Yes

☐ No

4. Do you run a home industry or business that is adversely affected due to rats eating your goods or supplies?

☐ Yes

☐ No

5. How much do rats or insects have a major impact on your daily activities (i.e., by eating your food, biting you or your children, etc.)?

☐ Very much

☐ A little

☐ None

Inadequate water access

1. Most of the time, how do you obtain water?

☐ Carrying containers to fetch water from someone else's tap or motor

☐ Hose brought to my home or lane by a water seller

☐ Private motor that pumps water directly to my home

2. How many times in a week do you get water? ________________

3. How much did your household spend on obtaining water in the last month? _________rupees

4. Has the time you spend getting water ever negatively impacted your work (for example, by causing you to be late for work, miss work, or come back early from work)?

☐ Yes

☐ No

5. Has the time spent getting water ever negatively impacted your children (for example, by causing them to be late for school, to miss school, or to lose time studying)?

☐ Yes

☐ No

Inadequate sanitation, solid waste, or electricity access

1. Where do you usually go to the bathroom?

☐ Open defecation

☐ Community or home toilet → Go to 1a and 1b

1a. If you use a toilet, do you use the toilet:

☐ For free

☐ I pay money

1b. Have you ever been late for or missed work because of time spent waiting in the queue for the community toilet?

☐ Yes

☐ No

Have you or your children ever faced the following problems while defecating in the open:

2. Faced tension when using the bathroom in the dark:

☐ Yes

☐ No

3. Hurt or injured yourself while toileting in the open:

☐ Yes

☐ No

4. Gotten harassed by other people while toileting:

☐ Yes

☐ No

5. Been harassed by the police because of toileting in the open:

☐ Yes

☐ No

6. Do you have your own official electricity meter provided by BEST?

☐ Yes, I have a BEST meter / Good → Go to 6a

☐No, I don't have a BEST meter but I have electricity / Medium→Go to 6b, 6c

☐ No, I don't have electricity / Bad → Go to 6c

6a. If you do have a meter, then do feel that you sometimes pay extra money in your electricity bill because someone else is stealing your electricity?

☐ Yes

☐ No

6b. If you don't have a meter, do you pay for electricity?

☐ Yes

☐ No

6c. If you don't have a meter or electricity, have you had tension because of trying to get an official BEST electricity meter unsuccessfully?

☐ Yes

☐ No

7. Have you ever had your electricity line cut or been fined by BEST?

☐ Yes

☐ No

Safety, crime and the police

1. During the time you have lived in Kaula Bandar, have you or has your family ever been robbed?

☐ Yes

☐ No

2. Have you or has anyone in your family gotten into a major physical fight with someone else in the community?

☐ Yes

☐ No

3. Have you or has anyone in your family ever been harassed by the police?

☐ Yes

☐ No

4. Have the police ever demanded money from you or a family member?

☐ Yes

☐ No

Migration

1. Were you born in Kaula Bandar, or did you migrate here?

☐ Born in Kaula Bandar

☐ Migrated to Kaula Bandar from a city area (including elsewhere in Mumbai) → Go to 1a

☐ Migrated to Kaula Bandar from a rural area → Go to 1a

1a. If a migrant to Kaula Bandar, how many years have you lived here?

_______________ years

Health conditions

1. Have you ever gone bankrupt because of spending on your health or a family member's health?

☐ Yes

☐ No

Income poverty

1. Have you ever had trouble getting a loan?

☐ Yes

☐ No

2. Do you actively have a loan out now?

☐ Yes → Go to 2a

☐ No

2a. If yes, are you having trouble repaying the loan?

☐ Yes

☐ No

3. Have you ever not had enough money to pay for your child's school enrollment?

☐ Yes

☐ No

4. How many times in the last month have you found yourself in a situation where you couldn't buy as much food as you needed for your family? __________

Occupation (men and women)

1. Are you employed?

☐ Yes → Go to 1a, 1b, 1c, 1d

☐ No

1a. If yes, are you the primary breadwinner?

☐ Yes

☐ No

1b. If you are employed, what is the occupation in which you spend the most time?

☐ Formal, stable job with the BMC or BPT

☐ Formal, stable job with a private company

☐ Contractual agreement with BMC or BPT

☐ Contractual agreement with private contractor

☐ Daily wager worker (i.e., casual construction worker)

☐ Have your own business (shopkeeper or home industry)

☐ Domestic worker (i.e., a maid)

1c. Have you ever had your salary cut due to illness or family emergency?

☐ Yes

☐ No

1d. Have you ever not been paid for work that you completed?

☐ Yes

☐ No

2. Have you felt that you haven't been able to get work because you're from a slum?

☐ Yes

☐ No

3. How many people earn money in your household? _____________

Gender-related adversities (women only)

1. Are you currently married?

☐ Yes → Go to 1a, 1b, 1c, 1d, 1e

☐ No

If yes, please answer the following questions?

1a. Does your husband allow you to leave your home and your lane without his permission?

☐ Yes

☐ No

1b. Have you ever had trouble getting employment because your husband won't give you permission?

☐ Yes

☐ No

1c. Do you face problems because your husband drinks alcohol or uses other drugs?

☐ Yes

☐ No

1d. Does your husband have another wife?

☐ Yes

☐ No

1e. Have you ever been physically been beaten by your husband or mother-in-law?

☐ Yes

☐ No

2. Do you have children?

☐ Yes → Go to 2a, 2b, 2c if woman is 35 years of age or younger

☐ No → Go to 2d if woman is 35 years of age or younger

If the woman is older than 35 years of age, stop the interview here!

2a. If you have delivered in the last 6 months, have you ever had trouble finding a place to rest in your home after delivering a child?

☐ Yes

☐ No

☐ Not applicable

2b. Do you have a boy child?

☐ Yes

☐ No → Go to 2c

2c. If you don't have a boy, do you have any tension because you haven't had a male child?

☐ Yes

☐ No

2d. If don't have a child, do you have any tension because you haven't had a child?

☐ Yes

☐ No

Section 6: Determining quantity of water use

We know water access is a very big problem in this community. We would like to get a sense of how much water you have used in the last week. To do this, we look at all the containers that you use to store water and ask you questions about them.

**Container**	**Capacity of the container in liters**	**How often did you fill this container with water in the last week?**	**Total Liters used**
Blue barrel no 1			
Blue barrel no 2			
Blue barrel no 3			
Container 1			
Container 2			
Container 3			
Container 4			
Container 5			
Container 6			
Container 7			
Container 8			
Container 9			
Container 10			
**Total Water Used in Last Week** (add values in last column) →			

CODING INTERPRETATION SHEET

Section 1: Observation Information

Variable 1= Date of survey
Variable 2= Interviewer's name
Variable 3= Respondent's name
Variable 4= Household Code

Variable 5= Home Condition by Observation
1=kutcha / Bad
2=semi-pucca / Medium
3=pucca / Good

Variable 6=Near to solid waste dump
1=Yes / Bad
2=No / Good

Variable 7=Near sea
1=Yes / Bad
2=No / Good

Variable 8=On the road
1=Yes / Bad
2=No / Good

Variable 9=Narrow lane
1=Yes / Bad
2=No / Good

Section 2: Demographics

Variable 10= Gender
1=Male
2=Female
3=Other

Variable 11=Age [Continuous]

Variable 12=Religion
1=Hindu
2=Muslim
3=Christian
4=Buddhist
5=Other → follow-up with Variable 12a

Variable 12a= name of other religion

Variable 13=Marital status

1=Never married
2=Currently married
3=Divorced
4=Husband or wife left me
5=Widowed

Variable 14=Region of origin
1=North Indian
2=South Indian
3=Maharashtrian
4=Bengal
5=Other → follow-up with Variable 14a

Variable 14a= name of other region of origin

Variable 15= Problem due to language
1=Yes / Bad
2= No / Good

Variable 16=Highest standard of school completed [Continuous variable]

Variable 17=Household income
1= <3000 rupees
2=3000-3999 rupees
3=4000-4999 rupees
4=5000-5999 rupees
5=6000-6999 rupees
6=7000-7999 rupees
7=8000-8999 rupees
8=9000-9999 rupees
9=10,000-10,999 rupees
10=11,000-11,999 rupees
11=>12,000 rupees → follow up with 17a
12=I don't know

Variable 17a= exact income specified if >12,000 rupees per month

Section 3: General Health Questionnaire

Variable 18= GHQ Q1 Concentration
0= Better than usual
1= Same as usual
2= Less than usual
3= Much less than usual

Variable 19= GHQ Q2 Sleep
0= Not at all
1= No more than usual
2= Rather more than usual

3= Much more than usual

Variable 20= GHQ Q3 Useful
0= More so than usual
1= Same as usual
2= Less useful than usual
3= Much less useful than usual

Variable 21= GHQ Q4 Decision-making
0= More so than usual
1= Same as usual
2= Less so than usual
3= Much less capable than usual

Variable 22= GHQ Q5 Strain
0= Not at all
1= No more than usual
2= Rather more than usual
3= Much more than usual

Variable 23= GHQ Q6 Overcoming difficulties
0= Not at all
1= No more than usual
2= Rather more than usual
3= Much more than usual

Variable 24= GHQ Q7 Enjoying activities
0= More so than usual
1= Same as usual
2= Less so than usual
3= Much less than usual

Variable 25= GHQ Q8 Facing problems
0= More so than usual
1= Same as usual
2= Less able than usual
3= Much less able than usual

Variable 26= GHQ Q9 Unhappy or depressed
0= Not at all
1= No more than usual
2= Rather more than usual
3= Much more than usual

Variable 27= GHQ Q10 Confidence
0= Not at all
1= No more than usual
2= Rather more than usual
3= Much more than usual

Variable 28= GHQ Q11 Worthless
0= Not at all
1= No more than usual
2= Rather more than usual
3= Much more than usual

Variable 29= GHQ Q12 Happy
0= More so than usual
1= About the same as usual
2= Less so than usual
3= Much less than usual

Variable 30= Likert Score

Variable 30a= GHQ Score

Variable 31= Do you believe that sadness or tension is an illness?
1= Yes
2= No

Variable 32= Do you believe this illness can be treated by a doctor?
1= Yes
2= No

Section 4: WHO Disability Assessment Schedule 2.0

Variable 33= Disability S1 Standing
1=None
2=Mild
3=Moderate
4=Severe
5=Extreme or cannot do

Variable 34= Disability S2 Household Responsibilities
1=None
2=Mild
3=Moderate
4=Severe
5=Extreme or cannot do

Variable 35= Disability S3 Learning a new task
1=None
2=Mild
3=Moderate
4=Severe
5=Extreme or cannot do

Variable 36= Disability S4 Community activities
1=None
2=Mild

3=Moderate
4=Severe
5=Extreme or cannot do

Variable 37= Disability S5 Emotionally affected
1=None
2=Mild
3=Moderate
4=Severe
5=Extreme or cannot do

Variable 38= Disability S6 Concentrating
1=None
2=Mild
3=Moderate
4=Severe
5=Extreme or cannot do

Variable 39= Disability S7 Walking
1=None
2=Mild
3=Moderate
4=Severe
5=Extreme or cannot do

Variable 40= Disability S8 Washing body
1=None
2=Mild
3=Moderate
4=Severe
5=Extreme or cannot do

Variable 41= Disability S9 Getting dressed
1=None
2=Mild
3=Moderate
4=Severe
5=Extreme or cannot do

Variable 42= Disability S10 Dealing with people
1=None
2=Mild
3=Moderate
4=Severe
5=Extreme or cannot do

Variable 43= Disability S11 Friendship
1=None
2=Mild
3=Moderate

4=Severe
5=Extreme or cannot do

Variable 44= Disability S12 Day-to-day work
1=None
2=Mild
3=Moderate
4=Severe
5=Extreme or cannot do

Variable 45= Disability Score

Variable 46= Disability H1 Days of difficulty [Continuous variable]

Variable 47= Disability H2 Unable to work [Continuous variable]

Variable 48= Disability H3 Cut back on activities [Continuous variable]

Variable 49= Trouble seeing
1=Yes
2=No

Variable 50= Trouble hearing
1=Yes
2=No

Variable 51= Difficulty with speech
1=Yes
2=No

Variable 52= Paralysis of body
1=Yes
2=No

Variable 53= Loss of limb
1=Yes
2=No

Section 5: Slum Adversity Questions

Overcrowding

Variable 54= Home spaces occupied [Continuous variable]

Variable 55= Number of people in home [Continuous variable]

Variable 56= Number of children <14 in home [Continuous variable]

Variable 57= Sleep outside
1= Yes / Bad
2= No / Good

Variable 58= Sleep sitting
1= Yes / Bad
2= No / Good

Variable 59= Conflict with neighbors
1= Yes / Bad
2= No / Good

Insecure residential status

Variable 60= Ownership status
1= Rented / Bad
2= Owned / Good

Variable 61= Ration card
1= Yes / Good → follow up with 61b, 61c, 61d
2= No / Bad → follow up with 61a

Variable 61a= If no to 61, then do get tension from trying to get a card?
1= Yes / Bad
2= No / Good

Variable 61b= If yes to 61, is name missing on card?
1= Yes / Bad
2= No / Good

Variable 61c= If yes to 61, is name mis-spelled?
1= Yes / Bad
2= No / Good

Variable 61d= If yes to 61, you are not getting enough kerosene or food?
1= Yes / Bad
2= No / Good

Variable 62= PAN Card
1= Yes / Good
2= No / Bad

Variable 63= Threatened with eviction
1= Yes / Bad
2= No / Good

Variable 64= Evicted by owner
1= Yes / Bad → follow up with 64a
2= No / Good

Variable 64a= Number of times evicted [Continuous variable]

Variable 65= Demolished by government
1= Yes / Bad → follow up with 65a
2= No / Good

Variable 65a= Number of times demolished [Continuous variable]

Poor structural quality of housing

Variable 66= Home burned down
1= Yes / Bad
2= No / Good

Variable 67= Small fire near home
1= Yes / Bad
2= No / Good

Variable 68= Home flooding
1= Yes / Bad
2= No / Good

Variable 69= Rats affecting business
1= Yes / Bad
2= No / Good

Variable 70= Rats affecting day-to-day activities
1= Very much / Bad
2= A little / Medium
3= None / Good

Inadequate water access

Variable 71= How do you get water
1= Carrying containers / Bad
2= Hose from water-seller / Medium
3= Private motor in home / Good

Variable 72= Number of times you get water [Continuous variable]

Variable 73= Water expenditure in last month [Continuous variable]

Variable 74= Negative impact from getting water
1= Yes / Bad
2= No / Good

Variable 75= Negative impact on children from water
1= Yes / Bad
2= No / Good

Inadequate access to toilets, solid waste, and electricity

Variable 76= Location of defecation
1= Open defecation / Bad
2= Toilet / Good → follow up with 76a and 76b

Variable 76a= Do you pay to toilet?
1= Free
2= Pay

Variable 76b= Late for work because of toilet
1= Yes / Bad
2= No / Good

Variable 77= Defecating in darkness
1= Yes / Bad
2= No / Good

Variable 78= Injury while toileting
1= Yes / Bad
2= No / Good

Variable 79= Harassed while toileting
1= Yes / Bad
2= No / Good

Variable 80= Harassed by police while toileting
1= Yes / Bad
2= No / Good

Variable 81= Electricity meter
1= Yes, have a meter / Good → follow up with 81a
2= No, no meter but have electricity / Medium → follow up with 81b, 81c
3= No, no electricity / Bad → follow up with 81c

Variable 81a= If yes, are you paying extra money?
1=Yes / Bad
2=No / Good

Variable 81b= If no, do you pay for electricity?
1= Yes / Bad
2= No / Good

Variable 81c= If no, have you had tension trying to get a meter?
1= Yes / Bad
2= No / Good

Variable 82= Electricity line has been cut
1= Yes / Bad
2= No / Good

Safety, crime, and the police

Variable 83= Victim of robbery
1= Yes / Bad
2= No / Good

Variable 84= Physical fight
1= Yes / Bad
2= No / Good

Variable 85= Police harassment
1= Yes / Bad
2= No / Good

Variable 86= Police demanded money
1= Yes / Bad
2= No / Good

Migration

Variable 87= Born or migrated to KB
1= Born in KB
2= Migrated from city → follow up with 87a
3= Migrated from rural area → follow up with 87a

Variable 87a= If migrated to KB, how many years in KB? [Continuous variable]

Health

Variable 88= Bankruptcy due to health
1=Yes /Bad
2=No / Good

Poverty

Variable 89= Difficulty getting loan
1=Yes /Bad
2=No / Good

Variable 90= Actively have loan
1=Yes /Bad → follow up with 90a
2=No / Good

Variable 90a= Trouble repaying loan
1=Yes /Bad
2=No / Good

Variable 91= Don't have money for school
1=Yes /Bad

2=No / Good

Variable 92= Number of times not able to buy food [Continuous variable]

Occupation

Variable 93= Employed?
1=Yes /Good → follow up with 93a, 93b, 93c, 93d
2=No / Bad

Variable 93a= Primary breadwinner?
1=Yes
2=No

Variable 93b= Occupation
1=Formal, stable job with the BMC or BPT
2=Formal, stable job with a private company
3=Contractual agreement with BMC or BPT
4=Contractual agreement with private contractor
5=Daily wager worker (i.e., casual construction worker)
6=Have your own business (shopkeeper or home industry)
7=Domestic worker (i.e., a maid)

Variable 93c= Salary cut
1= Yes / Bad
2= No / Good

Variable 93d= Not paid for work completed
1= Yes / Bad
2= No / Good

Variable 94= Not able to get work because from slum
1= Yes / Bad
2= No / Good

Variable 95= Number of earners in household [Continuous variable]

Gender-related questions (only for women)

Variable 96= Are you married?
1= Yes → follow up with 96a, 96b, 96c, 96d, 96e
2= No

Variable 96a= Able to leave home with husband's permission
1= Yes / Good
2= No / Bad

Variable 96b= Trouble getting employment because of husband
1= Yes / Bad
2= No / Good

Variable 96c= Husband uses alcohol or drugs
1= Yes / Bad
2= No / Good

Variable 96d= Husband has another wife
1= Yes / Bad
2= No / Good

Variable 96e= Domestic violence
1= Yes / Bad
2= No / Good

Variable 97= Do you have children?
1= Yes → follow up with 97a, 98, 99, 99a, 100
2= No

Variable 97a= Age <35
1= Yes
2= No

Variable 98= Trouble finding place to rest after delivery
1= Yes / Bad
2= No / Good
3= Not applicable

Variable 99= Boy child?
1= Yes
2= No

Variable 99a= Tension because of lack of boy child?
1= Yes / Bad
2= No / Good

Variable 100= Tension because of not having a child
1= Yes / Bad
2= No / Good
Variable 101=Total water used by household in last week [Continuous variable]
